# Influence of geometrical and operational parameters on tooth wear in the working mechanism of a satellite motor

**DOI:** 10.1038/s41598-023-44319-9

**Published:** 2023-10-09

**Authors:** Pawel Sliwinski

**Affiliations:** Division of Hydraulics and Pneumatics, Faculty of Mechanical Engineering and Ship Technology, Gdansk University of Technology, Ul. Gabriela Narutowicza 11/12, 80-233 Gdańsk, Poland

**Keywords:** Mechanical engineering, Engineering

## Abstract

This article describes the phenomena affecting the wear of the rotor of the working mechanism in a hydraulic satellite motor. The basic geometrical relationships that allow the calculation of the coordinates of the points of contact between the satellite and the rotor and the curvature are presented. A method for calculating the number of contacts of the satellite teeth with the rotor teeth and of the satellite teeth with the curvature teeth during one revolution of the rotor is proposed. A method of calculating the forces acting at the points of contact of the satellite with the rotor and the curvature is also proposed, as well as a method of calculating the stress in the tooth contact of the interacting components of the mechanism. The results of calculations of forces and stresses in tooth contact in a satellite mechanism consisting of a four-hump rotor and a six-hump curvature are presented. It is shown that the two chambers around the satellite are in the same phase in a certain range of the rotation angle of the rotor, i.e. in the emptying phase or in the filling phase. This results in the value of the force acting on the satellite resulting from the pressure difference being zero. It has also been shown that the most important parameters affecting tooth wear are the pressure difference in the working chambers of the satellite mechanism and the rotor speed.

## Introduction

The basic and main component in a hydrostatic drive system is the positive displacement pump. It is the component in the system where the greatest pressure increase occurs^1–5^. The second important component in the system is the rotary hydraulic motor. It is the displacement machine and the executive in the drive system. Its role is to convert hydraulic power into mechanical power. Today, there are many design variations of different types of hydraulic displacement machines on the world market—from piston machines to gear machines. They are used in the drive systems of various machines and even in laboratory test benches^6^. Work on the further development of the design of these machines and the improvement of their main operating parameters are still being carried out^6–11^. There are also attempts to describe the influence of various factors on the phenomena occurring in these machines^12–15^. Tests are also being conducted to verify the operation of these machines under various environmental conditions, such as thermal shocks^16–18^.

**Figure 1:**
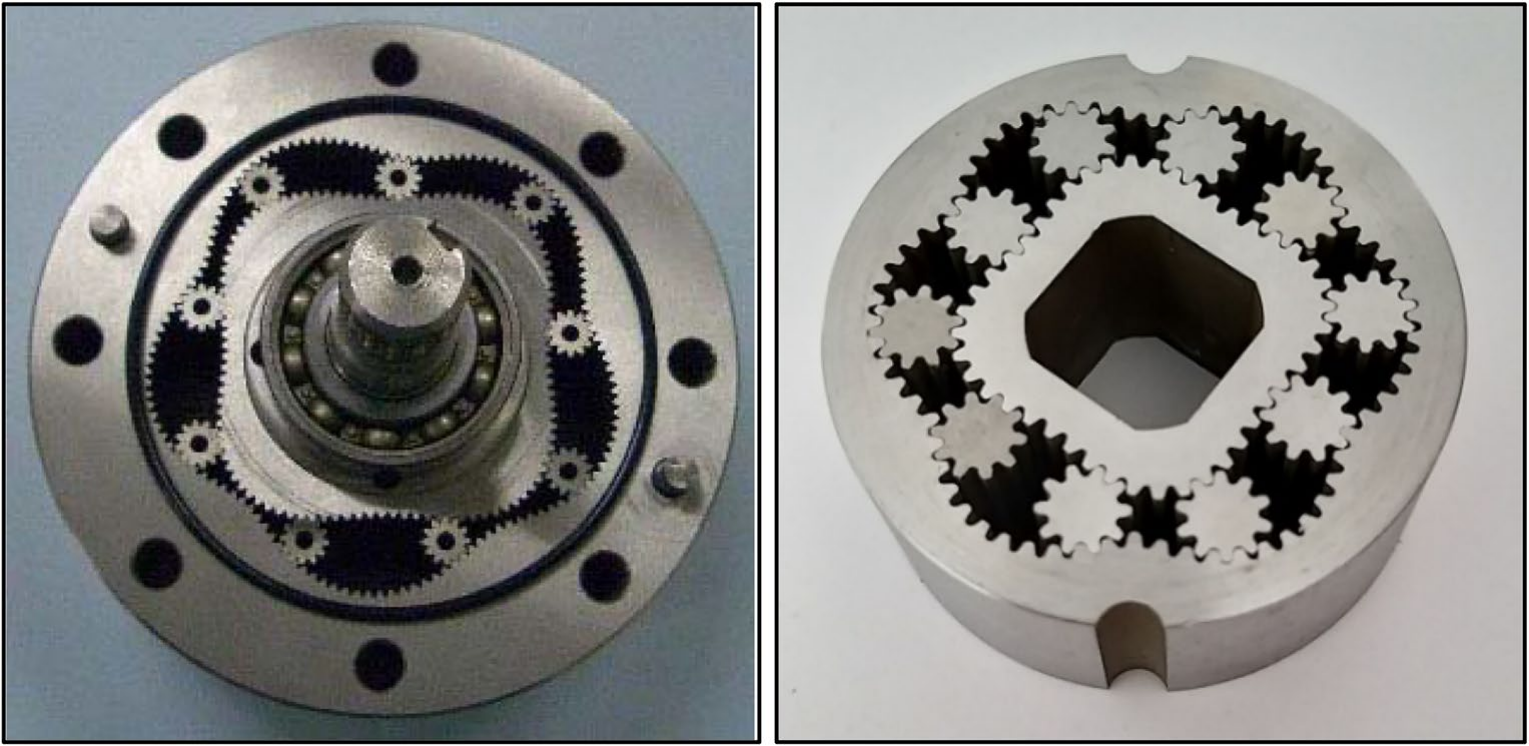
Examples of satellite mechanisms: type 4 × 5 (left) and type 4 × 6 (right)^19–21^.

Over the past 30 years, intensive design and development work has been carried out on innovative designs of gear displacement machines. These are machines whose working mechanism consists of non-circular toothed elements. This mechanism is commonly referred to as a satellite mechanism. Examples of satellite mechanisms as used in positive displacement machines are shown in Fig. 1.

The inner element of the satellite mechanism, mounted on a shaft, is called the rotor (or planet). The outer element is the curvature. The circular gear elements are the satellites. The photos above show only two types of mechanisms, namely the 4 × 5 and 4 × 6 types. The numbers in the type designation of the mechanism indicate the number of humps on the rotor and the number of humps on the curvature.

The following types of satellite mechanisms are known in the literature:

- type 2 × 2^22–26^;
- type 2 × 3^27–30^;
- type 2 × 4^22,24–26,30–34^;
- type 3 × 4^22,27,28,30,35–39^;
- type 4 × 5^20,21^;
- type 4 × 6^19,27,28,30,37,39–51^;
- type 6 × 8^19,39,51^;
- type 8 × 10^19,52,53^.

The methodology for designing these mechanisms can also be found in the above indicated literature, especially in Ref.^19^. Due to the fact that this literature is directly accessible (online), this methodology will not be summarized here. As already shown above, the satellite mechanism is a specific gear mechanism. The literature on gears and spur gears, regarding their design methods, kinematics and characteristics is also very extensive and widely available, for example in Refs.^54–58^. Literature on gearbox elements failures and their diagnosis are also available, for example in Refs.^59–61^. However, it is no less concerned with gears mounted on axles or shafts. That is, as in typical gears. The methodology for calculation the stresses in the teeth is also known for these gears and described in literature, for example in Refs.^57,62^. As can be seen in Fig. 1, the satellites in a satellite mechanism, are not fixed to an axle/shaft. The methodology for designing such mechanisms is already known^19,39^. On the other hand, there is a lack of sound knowledge on the method for calculation of the strength in the teeth of such mechanisms. Yes, methods for calculation the strength of teeth are known in the literature, but for gears with non-circular gears mounted on shafts. In Ref.^63^, for example, it was found that non-circular gear teeth are as strong in bending as circular gear teeth and for design purposes, classical spur gear theory can be used to predict static bending stresses. The analysis was carried out using a finite-element stress analysis. In Ref.^64^ an algorithm for mesh generation or the discretisation of gear tooth profiles of non-circular gear elements for static stress evaluation with FEM (Finite Element Method) was described. In Ref.^65^ the deformation of the teeth of a non-circular elliptical gear was analysed. It was found that the actual load on the teeth is variable and the deformation of cooperating pairs of teeth is also variable. This results from the smooth change in the gear ratio, which affects the change in the force acting on the individual teeth. Also in Refs.^66,67^ the mathematical formulae for analysis of the forces in planetary mechanism with eliptical gears were described. The planetary gear mechanism was also considered in Ref.^67^. In this publication the problem arising from the treatment of the pitch point force is described. In addition, the forces occurring in the gear and the efficiency of the gear are described, which is the main focus of the article.

The currently manufactured satellite mechanisms (especially 4 × 6) were designed to be made by chiselling^38,49,68,69^. In these mechanisms, the shape of the rotor consists of circular arcs with different radii and tangents to each other^39^. In this way, mechanisms with a tooth module of 1.5 mm were produced. Over time, manufacturers of satellite motors copied the shape of mechanisms made in this way and scaled them to other sizes, especially smaller sizes, with a smaller module of teeth. This allowed manufacturers to start production of other sizes of satellite motors^70,71^, with smaller working mechanisms using the wire electrical discharge machining (the so-called WEDM method).

## Object of research, research problem and the goal of analysis

**Figure 2:**
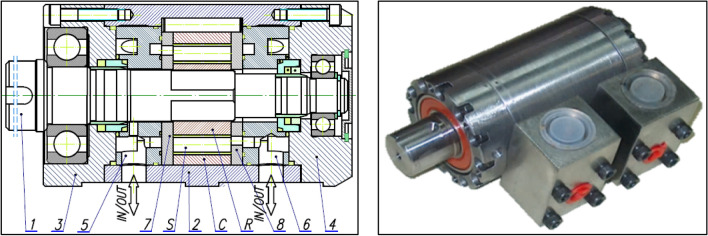
Prototype of the satellite motor: *R* rotor, *S* satellite, *C* curvatute, 1—shaft, 2,3 and 4—body, 5 and 6—manifolds, 7 and 8—commutation plate.

**Figure 3:**
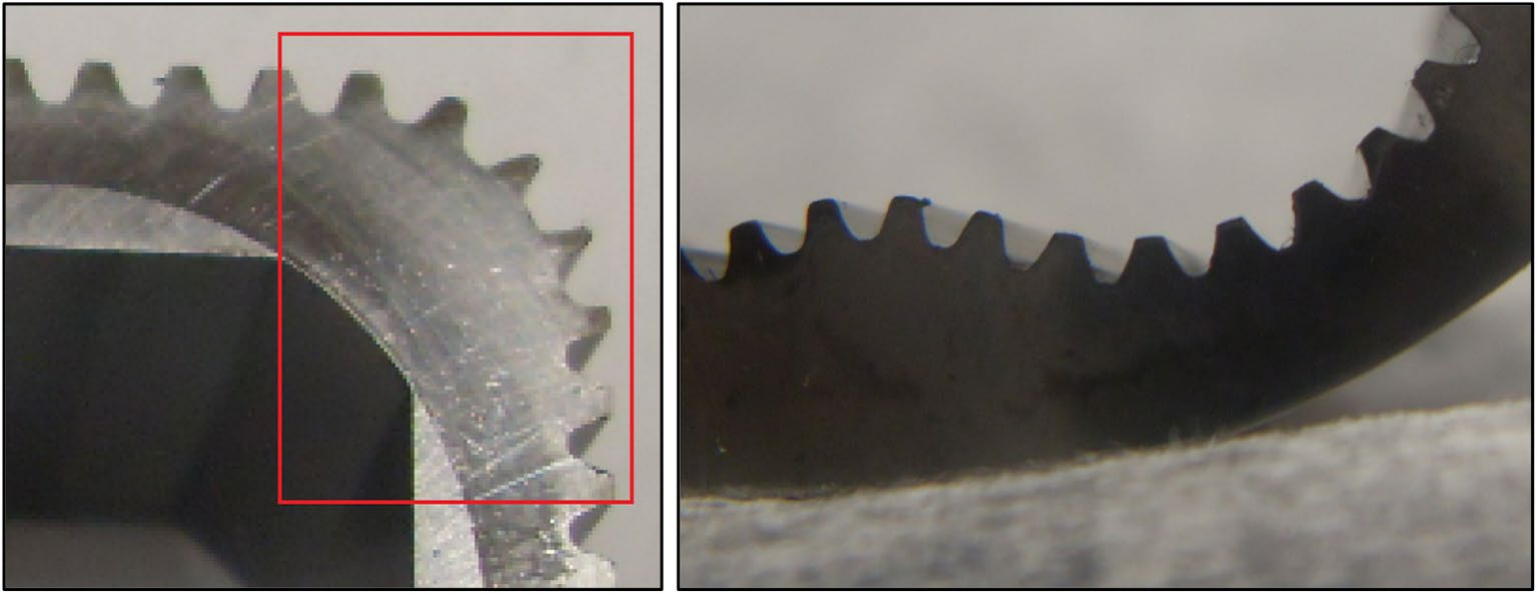
Specific wear of the teeth on the rotor hump (left)^19,30,37^. No apparent wear on the curvature teeth (right). Unknown operating time of the mechanism.

Unusual wear of the rotor’s teeth was noticed during laboratory testing of a prototype of hydraulic motor (Fig. 2) equipped with a 4 × 6 satellite mechanism. The specific wear occurs at the convexity (at the hump) of the rotor. However, no such wear was observed on the curvature teeth (Fig. 3).

Therefore, an attempt must be made to explain what influences such tooth wear. It is probably caused by exceeding the permissible pressures in the contact of the teeth of the interacting elements of the working mechanism. Therefore, it is advisable to analyze the loads acting on the teeth, which are influenced by the pressure difference acting on the satellite (resulting from the motor load and mechanical and pressure losses in the motor) and rotational speed of the rotor (motor shaft), resulting in inertial forces acting on the satellite. The number of contacts between the teeth of the rotor and the satellite and the number of contacts between the teeth of the curvature and the satellite also depends on the speed of rotation of the motor shaft. It is also advisable calculation the stresses in the contact of the teeth of the interacting elements and calculation of the permissible stresses in the contact of the teeth.

In order to calculate the loads acting on the teeth of the mechanism components, it is necessary to determine:

1. the basic geometrical relationships in the satellite mechanism;
2. the number of contacts of the satellite’s teeth with those of the rotor and the curvature during the rotation of the rotor;
3. the acceleration of the satellite as a function of the angle of rotation of the shaft (of the rotor);
4. pressures in the working chambers adjacent to the satellite.

Thus, as can be seen, the above-mentioned publication examples relate to various gears, the common feature of which is that the gears are mounted on an axle/shaft. Therefore, this article proposes an approximate method for calculating the stresses in the tooth contact of the satellite mechanism components. This makes it possible to estimate the value of these stresses and draw conclusions about the permissible motor load and speed. Currently, satellite engine manufacturers specify different motor speed ranges depending on the size of the engine. For example, in Refs.^70,71^ a speed range of 400 rpm to 2200 rpm is given. On the other hand, the nominal operating pressure for most satellite motors is given as 22 MPa and for some sizes as high as 25 MPa^70,71^. Therefore, the stress analyzes in the tooth contact are performed for a pressure difference in the working chambers of the motor of 25 MPa. For comparison purposes, stress values are also given for a small pressure difference, i.e. 5 MPa.

## Basic geometric relationships in the satellite mechanism

**Figure 4:**
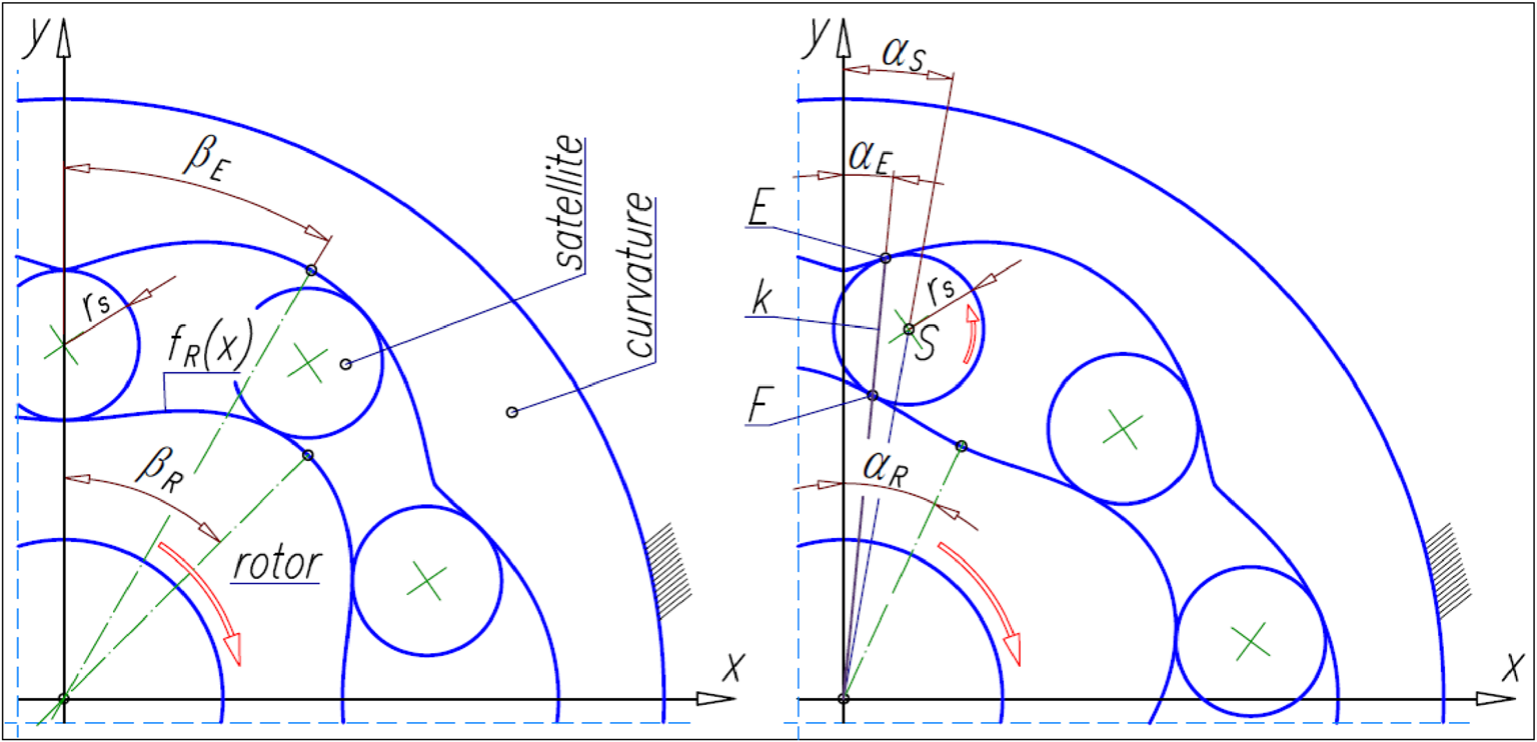
Satellite mechanism, its contact points and basic angles.

The methodology for designing satellite mechanisms is described in detail in Refs.^19,39,47^ and in Refs.^49,50^. These publications show that for any function f_R_(x) describing the rotor pitch line, it is possible to determine the coordinates of the curvature pitch line. The method for determining the curvature pitch line was described in detail in the publication^19^. The shape of the curvature pitch line and its mathematical notation depend on the number n_E_ of curvature humps. Regardless of the assumed number of curvature humps, the point E where the satellite touches the curvature lies on the line k passing through the rotor’s center of rotation and through the point F where the satellite touches the rotor^19^ (Fig. 4).

The coordinates of the point E are following:

1 $${\mathrm{x}}_{\mathrm{E}}={\mathrm{x}}_{\mathrm{F}}\cdot \left(\frac{{\mathrm{x}}_{\mathrm{F}}\cdot {\mathrm{x}}_{\mathrm{S}}+{\mathrm{y}}_{\mathrm{F}}\cdot {\mathrm{y}}_{\mathrm{S}}}{{{\mathrm{x}}_{\mathrm{F}}}^{2}+{{\mathrm{y}}_{\mathrm{F}}}^{2}}+\sqrt{{\left(\frac{{\mathrm{y}}_{\mathrm{F}}\cdot {\mathrm{y}}_{\mathrm{S}}+{\mathrm{x}}_{\mathrm{F}}\cdot {\mathrm{x}}_{\mathrm{S}}}{{{\mathrm{x}}_{\mathrm{F}}}^{2}+{{\mathrm{y}}_{\mathrm{F}}}^{2}}\right)}^{2}-\frac{\left({{\mathrm{x}}_{\mathrm{S}}}^{2}+{{\mathrm{x}}_{\mathrm{F}}}^{2}-{{\mathrm{r}}_{\mathrm{S}}}^{2}\right)}{{{\mathrm{x}}_{\mathrm{F}}}^{2}+{{\mathrm{y}}_{\mathrm{F}}}^{2}}}\right),$$

2 $${\mathrm{y}}_{\mathrm{E}}=\frac{{\mathrm{y}}_{\mathrm{F}}}{{\mathrm{x}}_{\mathrm{F}}}\cdot {\mathrm{x}}_{\mathrm{E}}.$$

Other basic relationships in the satellite mechanism are following:

- the central angle β_R_ covering one half of the cycle of the rotor pitch curve (corresponding to the length L_Rc_) is^19^:
  3 $${\upbeta }_{\mathrm{R}}=\frac{\uppi }{{\mathrm{n}}_{\mathrm{R}}},$$
  where n_R_ is the number of rotor humps;
- the central angle β_E_ covering one half of the cycle of the curvature pitch curve (corresponding to the length L_Ec_) is^19^:
  4 $${\upbeta }_{\mathrm{E}}=\frac{\uppi }{{\mathrm{n}}_{\mathrm{E}}},$$
  where n_E_ is the number of curvature humps;
- the angular position α_S_ of the satellite centre S corresponding to the angle α_R_ of the rotor rotation^19^:
  5 $${\mathrm{\alpha }}_{\mathrm{S}}=\frac{{\mathrm{n}}_{\mathrm{R}}}{{\mathrm{n}}_{\mathrm{E}}+{\mathrm{n}}_{\mathrm{R}}}\cdot {\mathrm{\alpha }}_{\mathrm{R}}.$$

**Figure 5:**
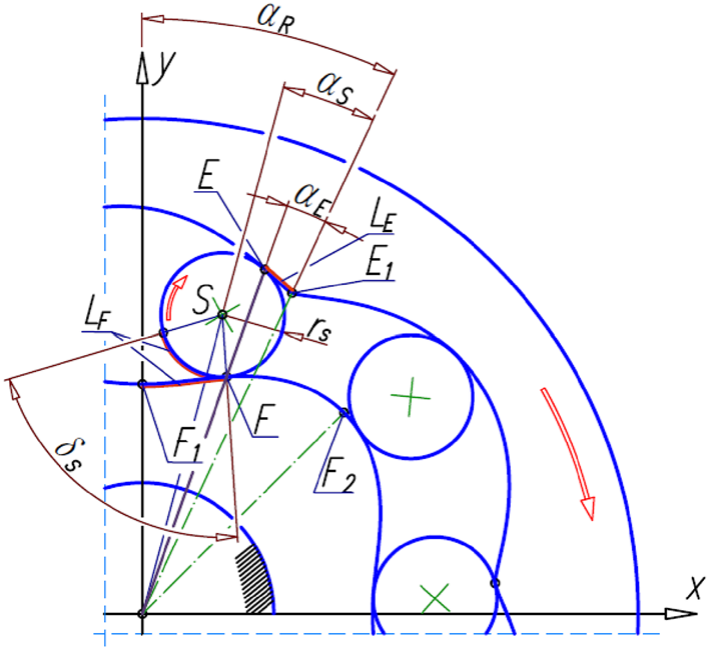
Lengths L_E_ and L_F_ and angle δ_S_ of satellite rotation.

During the rotation of the rotor by the angle α_R_, the satellite rolls around the rotor along the length L_F_ of the pitch line of the rotor (Fig. 5). So, the satellite rotates through the angle:

6 $${\updelta }_{\mathrm{S}}=\frac{{\mathrm{L}}_{\mathrm{F}}}{{\mathrm{r}}_{\mathrm{S}}},$$

where

7 $${\mathrm{L}}_{\mathrm{F}}={\int }_{{\mathrm{x}}_{\mathrm{F}1}}^{{\mathrm{x}}_{\mathrm{F}}}\sqrt{1+{\left(\frac{{\mathrm{df}}_{\mathrm{R}}(\mathrm{x})}{\mathrm{dx}}\right)}^{2}}\mathrm{dx},$$

8 $${\mathrm{r}}_{\mathrm{S}}=0.5\cdot \mathrm{m}\cdot {z}_{S},$$

and z_s_ is the number of teeth on the satellite.

The above relationship shows that in order to calculate the length L_F_ one must know the coordinates (x_F1_, y_F1_) and (x_F_, y_F_) of the points F_1_ and F. The coordinates of the point F_1_ are obvious and are: x_F1_ = 0 and y_F1_ = f_R_(0) (Fig. 5). However, to determine the coordinates (x_F_, y_F_) of the point F, it is necessary to know the coordinates (x_S_, y_S_) of the centre S of the satellite. The coordinates of the points F and S can be determined using the following system of equations:

9 $${\mathrm{y}}_{\mathrm{F}}={\mathrm{f}}_{\mathrm{R}}\left({\mathrm{x}}_{\mathrm{F}}\right),$$

10 $${\mathrm{y}}_{\mathrm{S}}=\mathrm{cot}{\mathrm{\alpha }}_{\mathrm{S}}\cdot {\mathrm{x}}_{\mathrm{S}},$$

11 $${\left({\mathrm{x}}_{\mathrm{F}}-{\mathrm{x}}_{\mathrm{S}}\right)}^{2}+{\left({\mathrm{y}}_{\mathrm{F}}-{\mathrm{y}}_{\mathrm{S}}\right)}^{2}={{\mathrm{r}}_{\mathrm{S}}}^{2},$$

12 $$\frac{{\mathrm{x}}_{\mathrm{S}}-{\mathrm{x}}_{\mathrm{F}}}{{\mathrm{y}}_{\mathrm{S}}-{\mathrm{y}}_{\mathrm{F }}}=\frac{\mathrm{d}}{\mathrm{dx}}{\mathrm{f}}_{\mathrm{R}}\left({\mathrm{x}}_{\mathrm{F}}\right).$$

The coordinates of the point F and centre S of the satellite are thus as follows:

13 $${\mathrm{x}}_{\mathrm{S}}=\sqrt{{{\mathrm{x}}_{\mathrm{F}}}^{2}+{\left({\mathrm{y}}_{\mathrm{F}}-{\mathrm{y}}_{\mathrm{S}}\right)}^{2}-{{\mathrm{r}}_{\mathrm{S}}}^{2}},$$

14 $${\mathrm{y}}_{\mathrm{S}}=\mathrm{cot}{\mathrm{\alpha }}_{\mathrm{S}}\cdot {\mathrm{x}}_{\mathrm{S}},$$

15 $${\mathrm{x}}_{\mathrm{F}}={\mathrm{f}}_{\mathrm{R}}^{-1}\left({\mathrm{y}}_{\mathrm{F}}\right),$$

16 $${\mathrm{y}}_{\mathrm{F}}={\mathrm{y}}_{\mathrm{S}}-\frac{{\mathrm{r}}_{\mathrm{S}}}{\sqrt{1+{\left(\frac{\mathrm{d}}{\mathrm{dx}}{\mathrm{f}}_{\mathrm{R}}\left({\mathrm{x}}_{\mathrm{F}}\right)\right)}^{2}}}\cdot \frac{\mathrm{d}}{\mathrm{dx}}{\mathrm{f}}_{\mathrm{R}}\left({\mathrm{x}}_{\mathrm{F}}\right).$$

The length L_F_ corresponds to the length L_E_ of the curvature pitch line (Fig. 5).

## The number of contacts of the teeth of the satellite with the teeth of the rotor and the curvature during the rotation of the rotor

**Figure 6:**
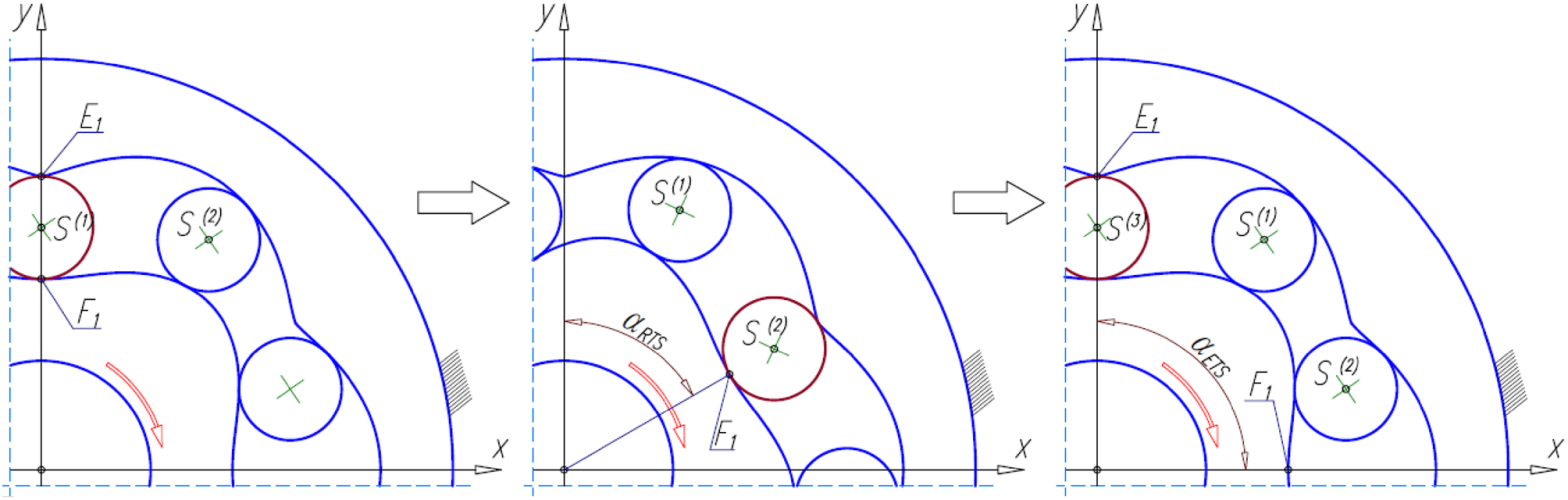
The contact point F_1_ of the tooth of the rotor with the tooth of the next satellite after the rotation of the rotor with the angle α_RTS_ and contact point E_1_ of the tooth of the curvature with the tooth of the next satellite after rotation of the rotor with the angle α_ETS_.

During the rotation of the rotor:

- each tooth of the rotor contacts the tooth of the next satellite (point F_1_ in Fig. 6) at each angle:
  17 $${\mathrm{\alpha }}_{\mathrm{RTS}}=2\uppi \cdot \frac{1}{{\mathrm{n}}_{\mathrm{E}}} \left[{\text{rad}}\right]={360}^{^\circ }\cdot \frac{1}{{\mathrm{n}}_{\mathrm{E}}} \left[{\text{deg}}\right],$$
- each tooth of the curvature contacts the tooth of the next satellite (point E_1_ in Fig. 6) at each angle:
  18 $${\mathrm{\alpha }}_{\mathrm{ETS}}=2\uppi \cdot \frac{1}{{\mathrm{n}}_{\mathrm{R}}} \left[{\text{rad}}\right]={360}^{^\circ }\cdot \frac{1}{{\mathrm{n}}_{\mathrm{R}}} \left[{\text{deg}}\right].$$

Thus, during one full revolution of the rotor, the number of contacts of each tooth of the rotor with the tooth of the satellites is:

19 $${\mathrm{i}}_{\mathrm{RTS}}=2\uppi \cdot \frac{1}{{\mathrm{\alpha }}_{\mathrm{RTS}}}={\mathrm{n}}_{\mathrm{E}},$$

while the number of contacts of each tooth of the curvature with the tooth of the satellites is:

20 $${\mathrm{i}}_{\mathrm{ETS}}=2\uppi \cdot \frac{1}{{\mathrm{\alpha }}_{\mathrm{ETS}}}={\mathrm{n}}_{\mathrm{R}}.$$

From the above formulas, it can be concluded that the following relationship exists between i_RTS_ and i_ETS_:

21 $${\mathrm{i}}_{\mathrm{RTS}}=\frac{{\mathrm{n}}_{\mathrm{E}}}{{\mathrm{n}}_{\mathrm{R}}}\cdot {\mathrm{i}}_{\mathrm{ETS}}.$$

After determining the coordinates (x_E_, y_E_) of points E on the curvature corresponding to the elementary angle δα_R_ of the rotor rotation, the length L_E_ can be calculated as the sum of the elementary sections of the curvature pitch line. The method of determining the length L_E_ is described in detail in the publication^1^. Using the lengths L_F_ and L_E_ the corresponding number of teeth can be calculated.

If the rotor rotates by the angle α_R_, then:

- the number z_RF_ of rotor teeth that were in contact with satellite teeth (the number is z_SF_) is:
  22 $${\mathrm{z}}_{\mathrm{RF}}={\mathrm{z}}_{\mathrm{SF}}=\frac{{\mathrm{L}}_{\mathrm{F}}}{\uppi \cdot \mathrm{m}},$$
- the number z_CE_ of curvature teeth that were in contact with satellite teeth (the number is z_SE_) is:
  23 $${\mathrm{z}}_{\mathrm{CE}}={\mathrm{z}}_{\mathrm{SE}}=\frac{{\mathrm{L}}_{\mathrm{E}}}{\uppi \cdot \mathrm{m}}.$$

If α_R_ = 360° then L_F_ is the length of the pitch line of the rotor, i.e.:

24 $${\mathrm{L}}_{\mathrm{F}\left({\mathrm{\alpha }}_{\mathrm{R}}={360}^{^\circ }\right)}=\uppi \cdot \mathrm{m}\cdot {\mathrm{z}}_{\mathrm{R}},$$

where z_R_ is the number of teeth of the rotor. Then from Eqs. (6) and (8) the angle of satellite rotation is:

25 $${\updelta }_{\mathrm{S}\left({\mathrm{\alpha }}_{\mathrm{R}}={360}^{^\circ }\right)}=2\cdot\uppi \cdot {\mathrm{i}}_{\mathrm{SR}} \left[{\text{rad}}\right]=360\cdot {\mathrm{i}}_{\mathrm{SR}} \left[{\text{deg}}\right],$$

where

26 $${\mathrm{i}}_{\mathrm{SR}}=\frac{{\mathrm{z}}_{\mathrm{R}}}{{\mathrm{z}}_{\mathrm{S}}},$$

is the number of revolutions of the satellite around its own axis and at the same time the number of contacts of each tooth of the satellite with the teeth of the rotor.

Equation (5) shows that the angular position of the satellite at one full revolution of the rotor is as follows:

27 $${\mathrm{\alpha }}_{\mathrm{S}\left({\mathrm{\alpha }}_{\mathrm{R}}={360}^{^\circ }\right)}=2\uppi \cdot \frac{{\mathrm{n}}_{\mathrm{R}}}{{\mathrm{n}}_{\mathrm{E}}+{\mathrm{n}}_{\mathrm{R}}} \left[{\text{rad}}\right]=360\cdot \frac{{\mathrm{n}}_{\mathrm{R}}}{{\mathrm{n}}_{\mathrm{E}}+{\mathrm{n}}_{\mathrm{R}}} \left[{\text{deg}}\right].$$

For this position of the satellite, there are points E and F. The coordinates of these points can be calculated using the formulas (1), (2) and (13) ÷ (16). Thus, if α_R_ = 360°, then the length of the pitch line of curvature is:

28 $${\mathrm{L}}_{\mathrm{E}\left({\mathrm{\alpha }}_{\mathrm{R}}={360}^{^\circ }\right)}=\uppi \cdot \mathrm{m}\cdot {\mathrm{z}}_{\mathrm{E}\left({\mathrm{\alpha }}_{\mathrm{R}}={360}^{^\circ }\right)},$$

where z_E_ is the number of teeth of the curvature, that were in contact with the teeth of the satellite during its roll along the curvature, i.e. z_E(_α_R = 360°)_ = z_S(_α_R = 360°)_. Thus, the number of revolutions of the satellite along the pitch line of the curvature is:

29 $${\mathrm{i}}_{\mathrm{SE}}=\frac{{\mathrm{z}}_{\mathrm{E}\left({\mathrm{\alpha }}_{\mathrm{R}}={360}^{^\circ }\right)}}{{\mathrm{z}}_{\mathrm{S}}} ,$$

and at the same time the number of contacts of each tooth of the satellite with the teeth of the curvature.

The total number of contacts of the teeth of the satellite with the teeth of the rotor and the curvature during one revolution of the rotor is:

30 $${\mathrm{i}}_{\mathrm{S}}={\mathrm{i}}_{\mathrm{SR}}+{\mathrm{i}}_{\mathrm{SE}}=\frac{{\mathrm{z}}_{\mathrm{R}}+{\mathrm{z}}_{\mathrm{E}\left({\mathrm{\alpha }}_{\mathrm{R}}={360}^{^\circ }\right)}}{{\mathrm{z}}_{\mathrm{S}}}.$$

If the satellite rolls along the entire length of the curvature pitch line, i.e. α_S_ = 360°, then the rotor makes the following number of revolutions:

31 $${\mathrm{i}}_{\mathrm{RE}}=360\cdot \frac{1}{{\mathrm{\alpha }}_{\mathrm{S}\left({\mathrm{\alpha }}_{\mathrm{R}}={360}^{\mathrm{o}}\right)}}=\frac{{\mathrm{n}}_{\mathrm{E}}}{{\mathrm{n}}_{\mathrm{R}}}+1.$$

## Loads on the teeth of the interacting elements of the satellite mechanism

In working hydraulic motor, the satellite mechanism is loaded by active and passive forces. The active forces result directly from the motor load with torque M. The effect of the motor load (torque M) is the pressure difference ∆p_i_ in the working chambers of the motor. This pressure difference generates forces that load the interacting teeth of the elements of the working mechanism. The passive forces are the effects of the rotational speed of the motor shaft. Thus, they are the inertial forces of the satellites. These forces also have a direct influence on the load of the teeth. To determine these forces, it is necessary to calculate the total acceleration of the satellite as a function of the angle of rotation of the rotor (the shaft).

### Accelerations of the satellite

**Figure 7:**
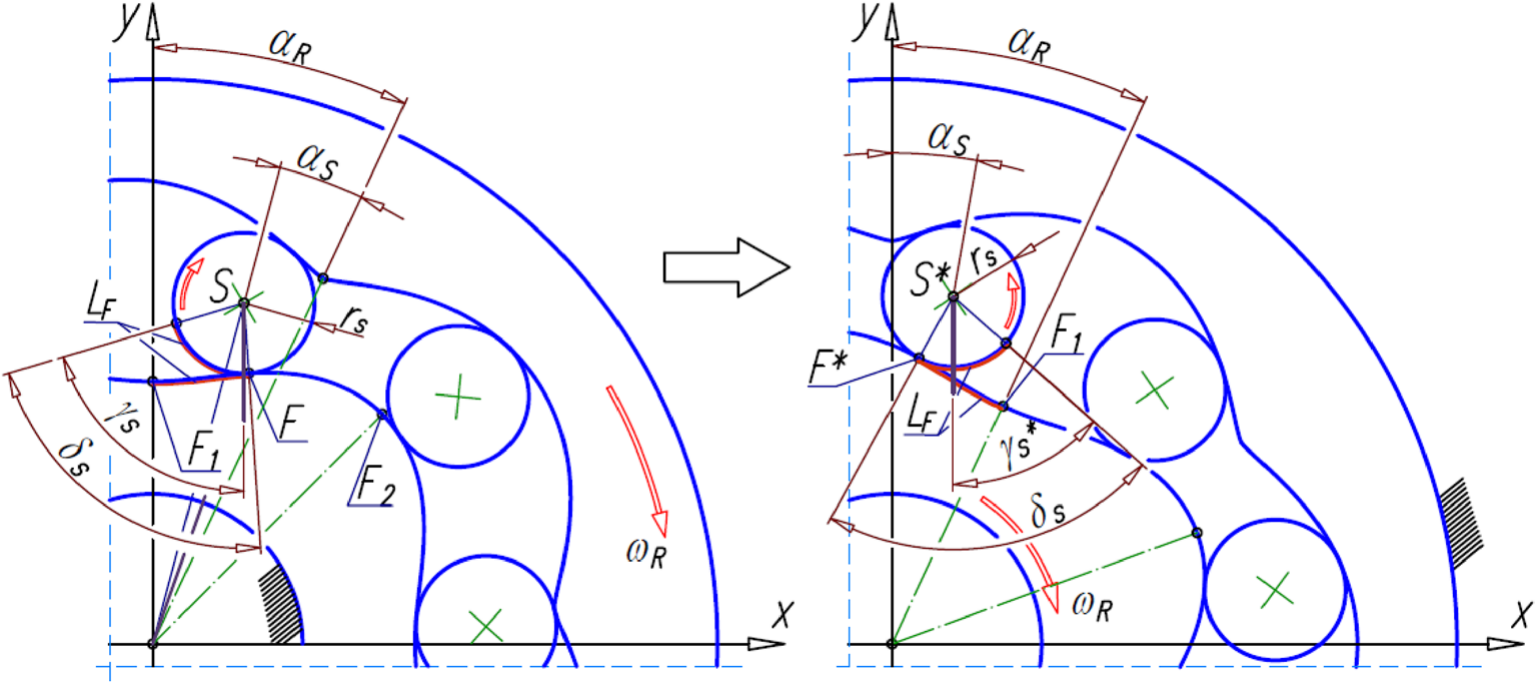
Transform the points E, F, S and the angle γ_S_ in a mechanism with a rotating curvature into the points E*, F*, S* and the angle γ_S_* in a mechanism with a rotating rotor.

In the satellite mechanism, the satellite moves in a flat motion. So there is a linear and an angular acceleration of the satellite. The angle γ_S_ is necessary to determine the angular acceleration of the satellite (Fig. 7). This angle is different for a mechanism with a rotating rotor than for a mechanism with a rotating curvature. Therefore, this angle is referred to as γ_S_* for a mechanism with a rotating rotor. The points S and F also become the points S* and F*.

The values of the angle γ_S_* can be calculated from the relation:

32 $${\upgamma }_{\mathrm{S}}^{*}={\updelta }_{\mathrm{S}}-\mathrm{atan}\left(\frac{{\mathrm{x}}_{\mathrm{S}}^{*}-{\mathrm{x}}_{\mathrm{F}}^{*}}{{\mathrm{y}}_{\mathrm{S}}^{*}-{\mathrm{y}}_{\mathrm{F}}^{*}}\right),$$

where x_S_*, y_S_*, x_F_*, x_F_* are the coordinates of the points S* and F*.

The linear acceleration of the satellite is:

33 $${\mathrm{a}}_{\mathrm{S}}=\frac{{\mathrm{d}}^{2}{\mathrm{L}}_{\mathrm{S}}^{*}}{{\mathrm{dt}}^{2}},$$

where L_S_^*^ is the distance between the centre S* of the satellite and the axis of rotation of the rotor, i.e.:

34 $${\mathrm{L}}_{\mathrm{S}}^{*}=\sqrt{{\left({\mathrm{x}}_{\mathrm{S}}^{*}\right)}^{2}+{\left({\mathrm{y}}_{\mathrm{S}}^{*}\right)}^{2}} ,$$

and the angular acceleration of the satellite is:

35 $${\upvarepsilon }_{\mathrm{S}}=\frac{{\mathrm{d}}^{2}{\upgamma }_{\mathrm{S}}^{*}}{{\mathrm{dt}}^{2}}.$$

If the angular velocity ω_R_ of the motor shaft (and therefore of the rotor) is constant (ω_R_ = const.), then:

36 $${\mathrm{a}}_{\mathrm{S}}={\upomega }_{\mathrm{R}}\cdot \frac{{\mathrm{d}}^{2}{\mathrm{L}}_{\mathrm{S}}^{*}}{\mathrm{d}{\mathrm{\alpha }}_{\mathrm{R}}^{2}} ,$$

37 $${\upvarepsilon }_{\mathrm{S}}={\upomega }_{\mathrm{R}}\cdot \frac{{\mathrm{d}}^{2}{\upgamma }_{\mathrm{S}}}{\mathrm{d}{\mathrm{\alpha }}_{\mathrm{R}}^{2}}.$$

### The centrifugal force acting on the satellite

**Figure 8:**
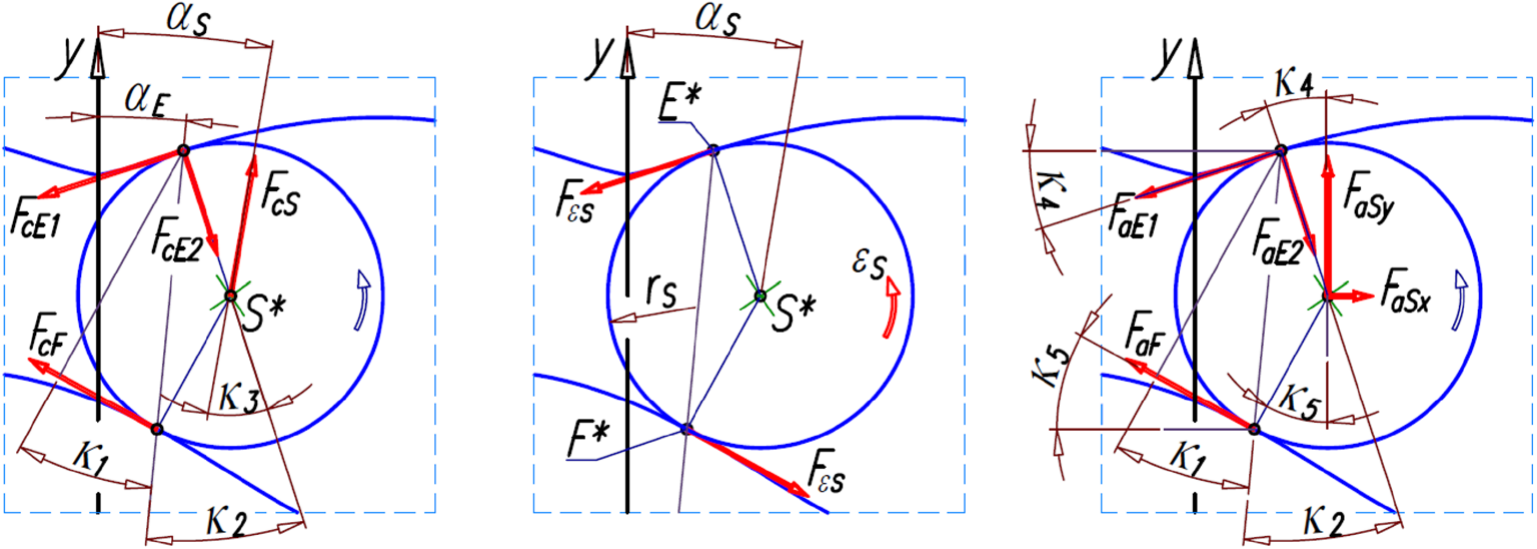
Forces acting on the satellite due to its plane motion.

The centrifugal force F_cS_ acts on the satellite (Fig. 8). If the angular velocity ω_R_ of the motor shaft (rotor) is constant (ω_R_ = const.), then:

38 $${\mathrm{F}}_{\mathrm{cS}}={\mathrm{m}}_{\mathrm{S}}\cdot {\upomega }_{\mathrm{R}}^{2}\cdot {\left(\frac{\mathrm{d}{\mathrm{\alpha }}_{\mathrm{S}}}{\mathrm{d}{\mathrm{\alpha }}_{\mathrm{R}}}\right)}^{2}\cdot {\mathrm{L}}_{\mathrm{S}}^{*}.$$

The effect of this force are the following forces in the contact points E* and F* (Fig. 8):

39 $${\mathrm{F}}_{\mathrm{cF}}={\mathrm{F}}_{\mathrm{cS}}\cdot \frac{{\mathrm{r}}_{\mathrm{S}}}{\sqrt{{\left({\mathrm{x}}_{\mathrm{E}}^{*}-{\mathrm{x}}_{\mathrm{F}}^{*}\right)}^{2}+{\left({\mathrm{y}}_{\mathrm{E}}^{*}-{\mathrm{y}}_{\mathrm{F}}^{*}\right)}^{2}}}\cdot \frac{\mathrm{sin}{\upkappa }_{3}}{\mathrm{sin}{\upkappa }_{1}} ,$$

40 $${\mathrm{F}}_{\mathrm{cE}1}={\mathrm{F}}_{\mathrm{cS}}\cdot \mathrm{sin}{\upkappa }_{3}+{\mathrm{F}}_{\mathrm{cF}}\cdot \mathrm{sin}\left({\upkappa }_{1}+{\upkappa }_{2}\right),$$

41 $${\mathrm{F}}_{\mathrm{cE}2}={\mathrm{F}}_{\mathrm{cS}}\cdot \mathrm{cos}{\upkappa }_{3}-{\mathrm{F}}_{\mathrm{cF}}\cdot \mathrm{cos}\left({\upkappa }_{1}+{\upkappa }_{2}\right),$$

where

42 $${\upkappa }_{1}=\mathrm{arctan}\left(\frac{{\mathrm{x}}_{\mathrm{S}}^{*}-{\mathrm{x}}_{\mathrm{F}}^{*}}{{\mathrm{y}}_{\mathrm{S}}^{*}-{\mathrm{y}}_{\mathrm{F}}^{*}}\right)-{\mathrm{\alpha }}_{\mathrm{E}},$$

43 $${\upkappa }_{2}=\mathrm{arctan}\left(\frac{{\mathrm{x}}_{\mathrm{S}}^{*}-{\mathrm{x}}_{\mathrm{E}}^{*}}{{\mathrm{y}}_{\mathrm{E}}^{*}-{\mathrm{y}}_{\mathrm{S}}^{*}}\right)+{\mathrm{\alpha }}_{\mathrm{E}},$$

44 $${\upkappa }_{3}=\mathrm{arctan}\left(\frac{{\mathrm{x}}_{\mathrm{S}}^{*}-{\mathrm{x}}_{\mathrm{E}}^{*}}{{\mathrm{y}}_{\mathrm{E}}^{*}-{\mathrm{y}}_{\mathrm{S}}^{*}}\right)+{\mathrm{\alpha }}_{\mathrm{S}}.$$

However, the coordinates of the points S*, F* and E* can be calculated from the following relations (Fig. 7):

45 $${\mathrm{x}}_{\mathrm{S}}^{*}=-\sqrt{{\mathrm{x}}_{\mathrm{S}}^{2}+{\mathrm{y}}_{\mathrm{S}}^{2}}\cdot \mathrm{sin}\left(\mathrm{arcsin}\left(\frac{{\mathrm{x}}_{\mathrm{S}}}{\sqrt{{\mathrm{x}}_{\mathrm{S}}^{2}+{\mathrm{y}}_{\mathrm{S}}^{2}}}\right)-{\mathrm{\alpha }}_{\mathrm{R}}\right),$$

46 $${\mathrm{y}}_{\mathrm{S}}^{*}=\sqrt{{\mathrm{x}}_{\mathrm{S}}^{2}+{\mathrm{y}}_{\mathrm{S}}^{2}}\cdot \mathrm{cos}\left(\mathrm{arcsin}\left(\frac{{\mathrm{x}}_{\mathrm{S}}}{\sqrt{{\mathrm{x}}_{\mathrm{S}}^{2}+{\mathrm{y}}_{\mathrm{S}}^{2}}}\right)-{\mathrm{\alpha }}_{\mathrm{R}}\right),$$

47 $${\mathrm{x}}_{\mathrm{F}}^{*}=-\sqrt{{\mathrm{x}}_{\mathrm{F}}^{2}+{\mathrm{y}}_{\mathrm{F}}^{2}}\cdot \mathrm{sin}\left(\mathrm{arcsin}\left(\frac{{\mathrm{x}}_{\mathrm{F}}}{\sqrt{{\mathrm{x}}_{\mathrm{F}}^{2}+{\mathrm{y}}_{\mathrm{F}}^{2}}}\right)-{\mathrm{\alpha }}_{\mathrm{R}}\right),$$

48 $${\mathrm{y}}_{\mathrm{F}}^{*}=\sqrt{{\mathrm{x}}_{\mathrm{F}}^{2}+{\mathrm{y}}_{\mathrm{F}}^{2}}\cdot \mathrm{cos}\left(\mathrm{arcsin}\left(\frac{{\mathrm{x}}_{\mathrm{F}}}{\sqrt{{\mathrm{x}}_{\mathrm{F}}^{2}+{\mathrm{y}}_{\mathrm{F}}^{2}}}\right)-{\mathrm{\alpha }}_{\mathrm{R}}\right),$$

49 $${\mathrm{x}}_{\mathrm{E}}^{*}={\mathrm{x}}_{\mathrm{F}}^{*}\cdot \left(\frac{{\mathrm{x}}_{\mathrm{F}}^{*}\cdot {\mathrm{x}}_{\mathrm{S}}^{*}+{\mathrm{y}}_{\mathrm{F}}^{*}\cdot {\mathrm{y}}_{\mathrm{S}}^{*}}{{\mathrm{x}}_{\mathrm{F}}^{2}+{\mathrm{y}}_{\mathrm{F}}^{2}}+\sqrt{{\left(\frac{{\mathrm{x}}_{\mathrm{F}}^{*}\cdot {\mathrm{x}}_{\mathrm{S}}^{*}+{\mathrm{y}}_{\mathrm{F}}^{*}\cdot {\mathrm{y}}_{\mathrm{S}}^{*}}{{\mathrm{x}}_{\mathrm{F}}^{2}+{\mathrm{y}}_{\mathrm{F}}^{2}}\right)}^{2}-\frac{{\mathrm{x}}_{\mathrm{S}}^{*2}+{\mathrm{y}}_{\mathrm{S}}^{*2}-{\mathrm{r}}_{\mathrm{S}}^{2}}{{\mathrm{x}}_{\mathrm{F}}^{2}+{\mathrm{y}}_{\mathrm{F}}^{2}}}\right),$$

50 $${\mathrm{y}}_{\mathrm{E}}^{*}=\frac{{\mathrm{y}}_{\mathrm{F}}^{*}}{{\mathrm{x}}_{\mathrm{F}}^{*}}\cdot {\mathrm{x}}_{\mathrm{E}}^{*}.$$

### Forces resulting from the angular and linear accelerations of the satellite

The effect of the angular acceleration ε_S_ and the linear acceleration α_S_ of the satellite is the force F_aS_ and the moment of force M_εS_, i.e.:

51 $${\mathrm{F}}_{\mathrm{aS}}={\mathrm{m}}_{\mathrm{S}}\cdot {\mathrm{a}}_{\mathrm{S}},$$

52 $${\mathrm{M}}_{\mathrm{\varepsilon S}}=\frac{1}{2}\cdot {\mathrm{m}}_{\mathrm{S}}\cdot {\mathrm{r}}_{\mathrm{S}}^{2}\cdot {\upvarepsilon }_{\mathrm{S}},$$

where m_S_ is the mass of the satellite. The moment M_εS_ can be replaced by the couple of forces F_εS_ (Fig. 8):

53 $${\mathrm{F}}_{\mathrm{\varepsilon S}}=\left({\mathrm{F}}_{\mathrm{\varepsilon SE}}={\mathrm{F}}_{\mathrm{\varepsilon SF}}\right)=\frac{1}{4}\cdot {\mathrm{m}}_{\mathrm{S}}\cdot {\mathrm{r}}_{\mathrm{S}}\cdot {\upvarepsilon }_{\mathrm{S}}.$$

The effect of the force F_εS_ are the following forces in the contact points E* and F*:

54 $${\mathrm{F}}_{\mathrm{aF}}=\frac{{\mathrm{F}}_{\mathrm{aSx}}\cdot \left({\mathrm{y}}_{\mathrm{E}}^{*}-{\mathrm{y}}_{\mathrm{S}}^{*}\right)+{\mathrm{F}}_{\mathrm{aSy}}\cdot \left({\mathrm{x}}_{\mathrm{S}}^{*}-{\mathrm{x}}_{\mathrm{E}}^{*}\right)}{\sqrt{{\left({\mathrm{x}}_{\mathrm{E}}^{*}-{\mathrm{x}}_{\mathrm{F}}^{*}\right)}^{2}+{\left({\mathrm{y}}_{\mathrm{E}}^{*}-{\mathrm{y}}_{\mathrm{F}}^{*}\right)}^{2}}}\cdot \frac{1}{\mathrm{cos}{\upkappa }_{1}},$$

55 $${\mathrm{F}}_{\mathrm{aE}1}={\mathrm{F}}_{\mathrm{aF}}\cdot \left(\mathrm{sin}{\upkappa }_{5}\cdot \mathrm{sin}{\upkappa }_{4}+\mathrm{cos}{\upkappa }_{5}\cdot \mathrm{cos}{\upkappa }_{4}\right)+{\mathrm{F}}_{\mathrm{aSx}}\cdot \mathrm{cos}{\upkappa }_{4}+{\mathrm{F}}_{\mathrm{aSy}}\cdot \mathrm{sin}{\upkappa }_{4},$$

56 $${\mathrm{F}}_{\mathrm{aE}2}={\mathrm{F}}_{\mathrm{aF}}\cdot \left(\mathrm{sin}{\upkappa }_{5}\cdot \mathrm{cos}{\upkappa }_{4}+\mathrm{cos}{\upkappa }_{5}\cdot \mathrm{sin}{\upkappa }_{4}\right)-{\mathrm{F}}_{\mathrm{aSx}}\cdot \mathrm{sin}{\upkappa }_{4}+{\mathrm{F}}_{\mathrm{aSy}}\cdot \mathrm{cos}{\upkappa }_{4},$$

where F_aSx_ and F_aSy_—components of the force F_aS_ responsible for the acceleration a_S_ (formula (33)), k_4_ and k_5_—angles (Fig. 8):

57 $${\upkappa }_{4}=\mathrm{arctan}\left(\frac{{\mathrm{x}}_{\mathrm{S}}^{*}-{\mathrm{x}}_{\mathrm{E}}^{*}}{{\mathrm{y}}_{\mathrm{E}}^{*}-{\mathrm{y}}_{\mathrm{S}}^{*}}\right),$$

58 $${\upkappa }_{5}=\mathrm{arctan}\left(\frac{{\mathrm{x}}_{\mathrm{S}}^{*}-{\mathrm{x}}_{\mathrm{F}}^{*}}{{\mathrm{y}}_{\mathrm{S}}^{*}-{\mathrm{y}}_{\mathrm{F}}^{*}}\right).$$

### The influence of the pressure difference in the working chambers on the load on the interacting teeth

In the satellite mechanism between the filling working chamber (high-pressure chamber HPC) and the emptying working chamber (low-pressure chamber LPC) exist pressure difference:

59 $$\Delta {\mathrm{p}}_{\mathrm{i}}={\mathrm{p}}_{\mathrm{H}}-{\mathrm{p}}_{\mathrm{L}},$$

where p_H_—high-pressure (in the filling working chamber), p_L_—low-pressure (in the emptying working chamber).

It should be noted that during the rotation of the rotor, the working chamber C passes from the high-pressure chamber HPC to the chamber with the maximum volume V_C-max_ and then to the low-pressure chamber LPC. Experimental studies have shown that in the V_C-max_ chamber there is a mean pressure p_M_ equal to (37) and (39):

60 $${\mathrm{p}}_{\mathrm{M}}=\frac{{\mathrm{p}}_{\mathrm{H}}+{\mathrm{p}}_{\mathrm{L}}}{2}.$$

**Figure 9:**
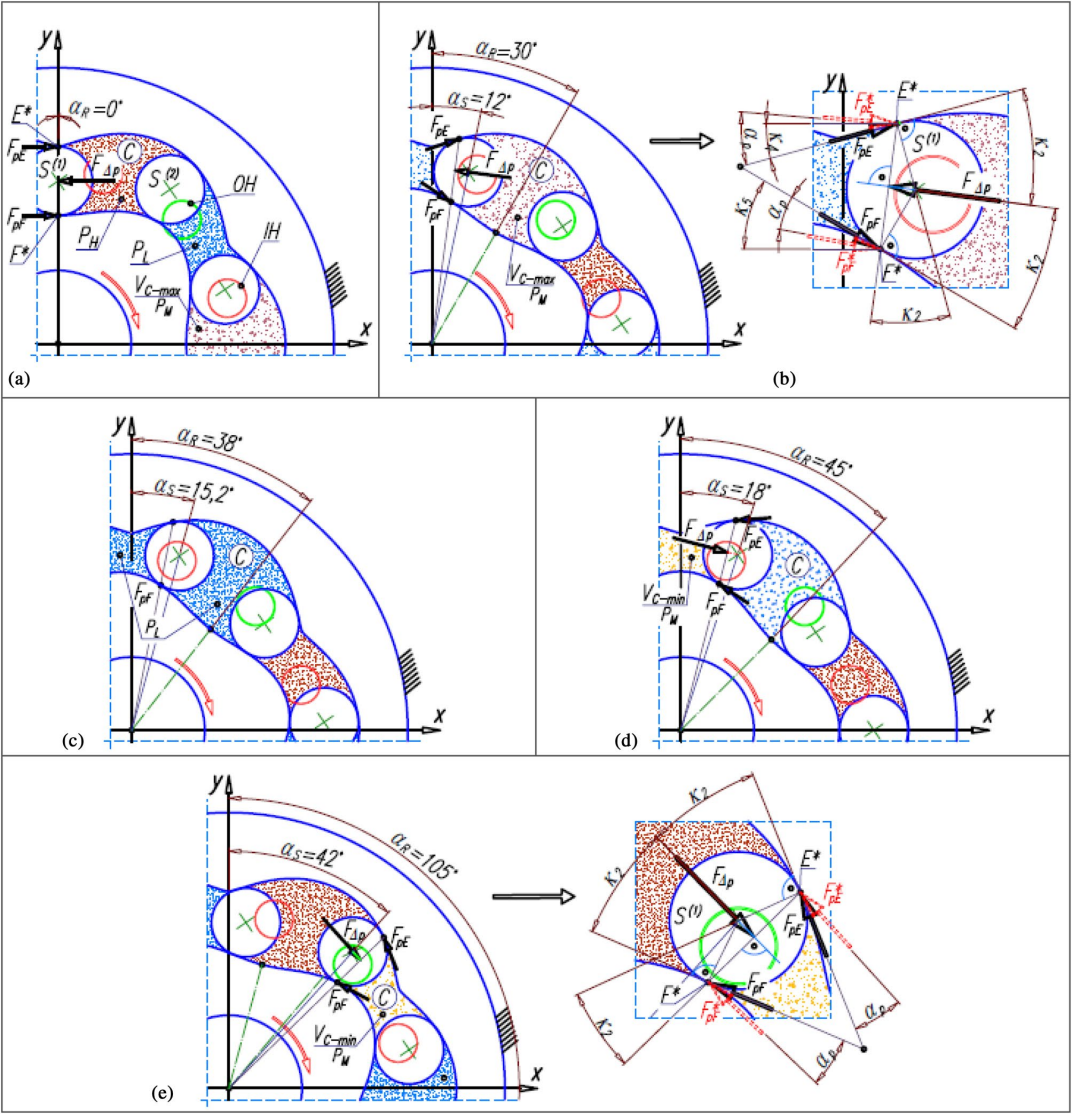
Forces originating from the pressure difference ∆p_i_ and acting on the satellite: (**a**) initial state (α_R_ = 0°); (**b**) example of the position of the working mechanism elements corresponding to two adjacent working chambers being in the same phase (emptying); (**c**) the position of the elements of the working mechanism corresponding to the chamber with the maximum volume V_C-max_; (**d,e**) the position of the elements of the working mechanism corresponding to the chamber with the minimum volume V_C-min_.

Thus, the value of the force F_∆p_ and its direction of action change abruptly (Fig. 9). In the satellite mechanism, the change of the volume of the working chamber C from V_C-max_ to V_C-min_ and vice versa take place at each angle of rotation of the rotor equal to:

61 $${{\mathrm{\alpha }}_{\mathrm{R}}}^{\left(\mathrm{C}\right)}=180\cdot \left(\frac{1}{{\mathrm{n}}_{\mathrm{E}}}+\frac{1}{{\mathrm{n}}_{\mathrm{R}}}\right).$$

The effect of the pressure difference ∆p_i_ is the pressure difference ∆p_S_ acting on the satellite. Thus, for:

- ${\mathrm{\alpha }}_{\mathrm{R}}\in \langle \left.0;\frac{180}{{\mathrm{n}}_{\mathrm{E}}}\right)$ and ${\mathrm{\alpha }}_{\mathrm{R}}\in \left(\frac{180}{{\mathrm{n}}_{\mathrm{R}}};\frac{180}{{\mathrm{n}}_{\mathrm{E}}}+{{\mathrm{\alpha }}_{\mathrm{R}}}^{\left(\mathrm{C}\right)}\right)$ is ∆p_S_ = ∆p_i_ and respectively:
  62 $${\mathrm{F}}_{\Delta \mathrm{p}}={\Delta \mathrm{p}}_{\mathrm{i}}\cdot \mathrm{H}\cdot \sqrt{{\left({\mathrm{x}}_{\mathrm{E}}^{*}-{\mathrm{x}}_{\mathrm{F}}^{*}\right)}^{2}+{\left({\mathrm{y}}_{\mathrm{E}}^{*}-{\mathrm{y}}_{\mathrm{F}}^{*}\right)}^{2}},$$
  where H is the height of the satellite mechanism;
- ${\mathrm{\alpha }}_{\mathrm{R}}\in \left(\frac{180}{{\mathrm{n}}_{\mathrm{E}}};\frac{180}{{\mathrm{n}}_{\mathrm{R}}}\right)$ and ${\mathrm{\alpha }}_{\mathrm{R}}\in \left(\frac{180}{{\mathrm{n}}_{\mathrm{E}}}+{{\mathrm{\alpha }}_{\mathrm{R}}}^{\left(\mathrm{C}\right)};\frac{180}{{\mathrm{n}}_{\mathrm{R}}}+{{\mathrm{\alpha }}_{\mathrm{R}}}^{\left(\mathrm{C}\right)}\right)$ two adjacent chambers are in the same phase, i.e. in the emptying phase or in the filling phase (Fig. 9c)). Therefore ∆p_S_ = 0 and F_∆p_ = 0, F_pE_ = 0, F_pF_ = 0 (see below);
- ${\mathrm{\alpha }}_{\mathrm{R}}=\frac{180}{{\mathrm{n}}_{\mathrm{E}}}$ and ${\mathrm{\alpha }}_{\mathrm{R}}=\frac{180}{{\mathrm{n}}_{\mathrm{E}}}+{{\mathrm{\alpha }}_{\mathrm{R}}}^{\left(\mathrm{C}\right)}$ and for ${\mathrm{\alpha }}_{\mathrm{R}}=\frac{180}{{\mathrm{n}}_{\mathrm{R}}}$:
  63 $$\Delta {\mathrm{p}}_{\mathrm{S}}=\frac{{\mathrm{p}}_{\mathrm{H}}-{\mathrm{p}}_{\mathrm{L}}}{2},$$
  and the F_∆p_ = should be calculated according to the formula (62) with the ∆p_S_ instead of ∆p_i_.

The circumferential forces F_pE_ and F_pF_, occurring at points E* and F* were assumed to be reactions to the force F_∆p_ (Fig. 9). The values of these forces can be calculated using the following relationship:

64 $${\mathrm{F}}_{\mathrm{pE}}={\mathrm{F}}_{\mathrm{pF}}=\frac{{\mathrm{F}}_{\Delta \mathrm{p}}}{2}\cdot \frac{\mathrm{cos}{\mathrm{\alpha }}_{\mathrm{p}}}{\mathrm{cos}\left({\upkappa }_{2}-{\mathrm{\alpha }}_{\mathrm{p}}\right)},$$

where α_p_ is the pressure angle.

### Total values of forces loading the interacting teeth of the mechanism elements

The total values of the circumferential forces F_pE_ and F_pF_ acting at points E* and F* are as follows:

- in the range of the rotor rotation angle ${\mathrm{\alpha }}_{\mathrm{R}}\in \langle \left.0;\frac{180}{{\mathrm{n}}_{\mathrm{E}}}\right)$:
  at point E*:
    65 $${\mathrm{F}}_{\mathrm{E}-\mathrm{p}}={{\mathrm{F}}_{\mathrm{pE}}-\mathrm{F}}_{\mathrm{aE}1}-{\mathrm{F}}_{\mathrm{cE}1}-{\mathrm{F}}_{\mathrm{\varepsilon SE}},$$
  at point F*:
    66 $${\mathrm{F}}_{\mathrm{F}-\mathrm{p}}={{\mathrm{F}}_{\mathrm{pF}}-\mathrm{F}}_{\mathrm{aF}}-{\mathrm{F}}_{\mathrm{cF}}+{\mathrm{F}}_{\mathrm{\varepsilon SF}},$$
- in the range of the rotor rotation angle ${\mathrm{\alpha }}_{\mathrm{R}}\in \langle \frac{180}{{\mathrm{n}}_{\mathrm{E}}};\frac{180}{{\mathrm{n}}_{\mathrm{R}}}\rangle$ and ${\mathrm{\alpha }}_{\mathrm{R}}\in \langle \frac{180}{{\mathrm{n}}_{\mathrm{E}}}+{{\mathrm{\alpha }}_{\mathrm{R}}}^{\left(\mathrm{C}\right)};\frac{180}{{\mathrm{n}}_{\mathrm{R}}}+{{\mathrm{\alpha }}_{\mathrm{R}}}^{\left(\mathrm{C}\right)}\rangle$:
  at point E*:
    67 $${\mathrm{F}}_{\mathrm{E}-\mathrm{p}}={\mathrm{F}}_{\mathrm{aE}1}+{\mathrm{F}}_{\mathrm{cE}1}+{\mathrm{F}}_{\mathrm{\varepsilon SF}},$$
  at point F*:
    68 $${\mathrm{F}}_{\mathrm{F}-\mathrm{p}}={\mathrm{F}}_{\mathrm{aF}}{+\mathrm{F}}_{\mathrm{cF}}-{\mathrm{F}}_{\mathrm{\varepsilon SF}},$$
- for $${\mathrm{\alpha }}_{\mathrm{R}}\in \left(\frac{180}{{\mathrm{n}}_{\mathrm{R}}};\frac{180}{{\mathrm{n}}_{\mathrm{E}}}+{{\mathrm{\alpha }}_{\mathrm{R}}}^{\left(\mathrm{C}\right)}\right)$$:
  at point E*:
    69 $${\mathrm{F}}_{\mathrm{E}-\mathrm{p}}={{\mathrm{F}}_{\mathrm{pE}}+\mathrm{F}}_{\mathrm{aE}1}+{\mathrm{F}}_{\mathrm{cE}1}+{\mathrm{F}}_{\mathrm{\varepsilon SE}},$$
  at point F*:
    70 $${\mathrm{F}}_{\mathrm{F}-\mathrm{p}}={{\mathrm{F}}_{\mathrm{pF}}+\mathrm{F}}_{\mathrm{aF}}+{\mathrm{F}}_{\mathrm{cF}}-{\mathrm{F}}_{\mathrm{\varepsilon SF}}.$$

## Problems of contact stress of the interacting teeth

**Figure 10:**
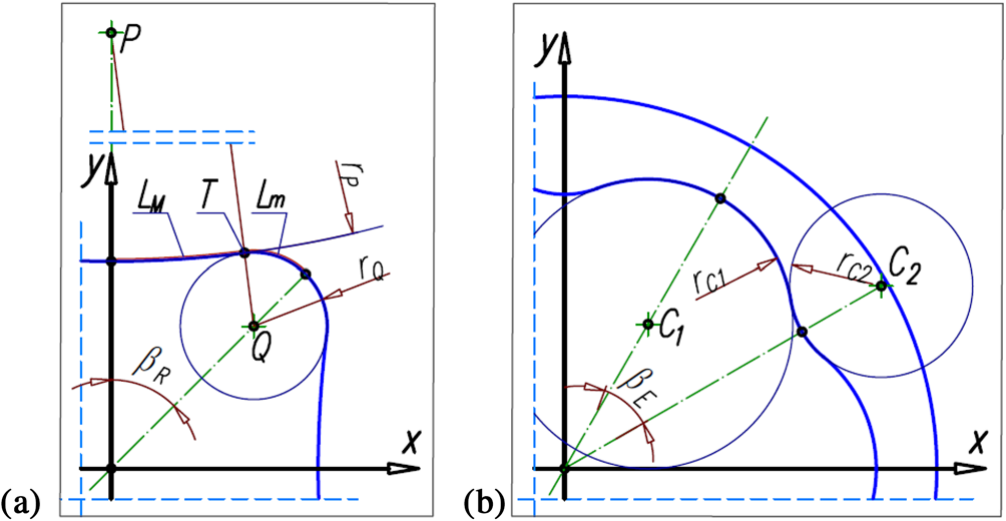
Tip clearance TC and circumferential backlash CB in satellite–rotor pair.

The method of calculating the permissible normal stresses σ_N_ in the contact of the interacting teeth of two steel gears is generally known and can be found, for example, in Ref.^57^. At the present stage of consideration, it is proposed to adopt the well-known model of permissible stresses (for gears) for the satellite mechanism. Because the radii of the pitch line of the rotor and the curvature are variable, it is proposed to take their smallest values for the strength calculations (i.e. r_Q_ and r_C2_ according to Fig. 10). Thus, for the teeth of the interacting elements, the contact ratio ε (according to Ref.^57^) is propose to calculate according to the following relation:

71 $$\upvarepsilon =\frac{\sqrt{{{\mathrm{r}}_{\mathrm{aS}}}^{2}-{{\mathrm{r}}_{\mathrm{bS}}}^{2}}+\sqrt{{{\mathrm{r}}_{\mathrm{a}}}^{2}-{{\mathrm{r}}_{\mathrm{b}}}^{2}}-{\mathrm{a}}_{\mathrm{w}}\cdot \mathrm{sin}{\mathrm{\alpha }}_{\mathrm{w}}}{{\mathrm{p}}_{\mathrm{bS}}},$$

where r_aS_—the tip radius of the satellite teeth, r_bS_—the base radius of the satellite, p_bS_—the base pitch of the satellite teeth, r_a_ and r_b_—the tip radius and the base radius of the circle of radius r_Q_ or r_C2_, a_w_—the true distance between the centre of the satellite and the circle of radius r_Q_ or the circle of radius r_C2_, α_w_—the pitch pressure angle.

For the gear elements of the satellite mechanism which are made of the same material, it is proposed to calculate the normal stress at the contact surface of the teeth according to the following formula:

72 $${\upsigma }_{\mathrm{N}}=\sqrt{\frac{4-\upvarepsilon }{6\uppi }\cdot \left(1+\frac{\mathrm{z}}{{\mathrm{z}}_{\mathrm{S}}}\right)\cdot \frac{\mathrm{F}}{\mathrm{H}\cdot {\mathrm{r}}_{\mathrm{S}}}\cdot \frac{\mathrm{E}}{1-{\upnu }^{2}}\cdot \frac{\mathrm{K}}{\mathrm{sin}{\mathrm{\alpha }}_{\mathrm{w}}\cdot \mathrm{cos}{\mathrm{\alpha }}_{\mathrm{p}}}} \le {\upsigma }_{\mathrm{per}},$$

where F—the circumferential force at the point of contact of the teeth of the mating elements, H—the length of the tooth line (the height of the satellite mechanism), E—the Young’s modulus of the gear material, ν—the Poisson’s ratio of the gear material, z—the theoretical number of teeth of the gear engaging the satellite, i.e. z_R_ or z_E_ (Fig. 10), K—the coefficient defined as^57,62^:

73 $$\mathrm{K}={\mathrm{K}}_{\mathrm{A}}\cdot {\mathrm{K}}_{\mathrm{v}}\cdot {\mathrm{K}}_{\mathrm{H}},$$

where K_A_—the overload factor, K_H_—the coefficient of uneven load distribution along the length of the tooth, Kv—the coefficient of dynamic force, σ_per_—permissible stress.

According to Refs.^57,62^, the value of permissible stress in gears is calculated as follows:

74 $${\upsigma }_{\mathrm{per}}=\frac{{\upsigma }_{\mathrm{Hlim}}}{{\mathrm{S}}_{\mathrm{H}}}\cdot \mathrm{Z},$$

where

75 $$\mathrm{Z}={\mathrm{Z}}_{\mathrm{NT}}\cdot {\mathrm{Z}}_{\mathrm{L}}\cdot {\mathrm{Z}}_{\mathrm{R}}\cdot {\mathrm{Z}}_{\mathrm{v}}\cdot {\mathrm{Z}}_{\mathrm{W}}\cdot {\mathrm{Z}}_{\mathrm{X}},$$

where σ_Hlim_—the contact fatigue strength of gears materials, Z_NT_—the coefficient of durability at contact load, Z_L_—the coefficient of oil viscosity, Z_R_—the factor of surface condition (roughness), Zv—the speed factor, Z_W_—the coefficient of surface squeeze (deformation), Z_X_—the size factor at contact loading, S_H_—the safety factor at contact loading.

In the literature (e.g.^57,62^) methods for estimating the values of the above coefficients of K and Z can be found, but for typical gears, i.e. for circular interacting toothed elements. The ranges of values of these coefficients and the safety factor are given in Table 1.

**Table 1:** The values of the coefficients type K and Z for nitrided elements^57^.

K_A_	K_H_	K_V_	S_H_	Z_NT_	Z_L_⋅Z_R_⋅Z_V_	Z_W_	Z_X_
1 ÷ 2.25	1 ÷ 1.25	1 ÷ 2.5	1 ÷ 1.3	0.85 ÷ 1.6	1.0	1.1	1.02

**Figure 11:**
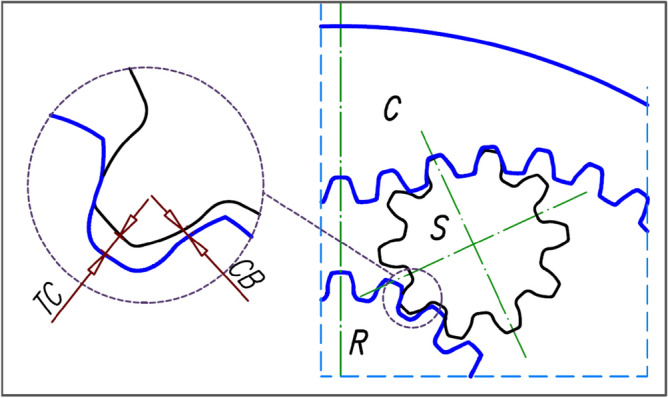
Geometry of the rotor of the tested satellite mechanism; r_C1_ and r_C2_—radii of the equivalent circles of curvature (for calculating the number of the contact ratio ε).

In the satellite mechanism, the satellite is not fixed on any axis. In this way, the satellite can change its position relative to the rotor and the curvature in terms of inter-tooth clearance and tip clearance. In extreme cases (with a suitable value of the tooth clearance), the tip clearance can be cancelled. Thus, the distance between the centre of the satellite and the centre of a circle of radius r_Q_ or r_C2_ can vary. In extreme cases, as a result of the centrifugal forces acting on the satellite, the circumferential backlash (and even the tip clearance) in the satellite-curvature pair may be erased and the circumferential backlash and the tip clearance in the satellite-rotor pair may increase (Fig. 11).

Thus, it can be assumed (especially in working gear) that the distance between the center of the satellite and:

- the centre of the circle of the rotor of radius r_Q_ is:
  76 $${\mathrm{a}}_{\mathrm{wQ}}={\mathrm{r}}_{\mathrm{S}}+{\mathrm{r}}_{\mathrm{Q}}-{\mathrm{h}}_{\mathrm{a}}+{\mathrm{h}}_{\mathrm{f}},$$
  where h_a_ and h_f_ are the height of the tooth head and the tooth foot, respectively;
- the centre of the circle of the rotor of radius r_P_ is:
  77 $${\mathrm{a}}_{\mathrm{wP}}={\mathrm{r}}_{\mathrm{S}}+{\mathrm{r}}_{\mathrm{P}}-{\mathrm{h}}_{\mathrm{a}}+{\mathrm{h}}_{\mathrm{f}},$$
- the centre of the circle of the curvature of radius r_C1_ is:
  78 $${\mathrm{a}}_{\mathrm{wC}1}={\mathrm{r}}_{\mathrm{S}}+{\mathrm{r}}_{\mathrm{C}1}+{\mathrm{h}}_{\mathrm{a}}-{\mathrm{h}}_{\mathrm{f}},$$
- the centre of the circle of the curvature of radius r_C2_ is:
  79 $${\mathrm{a}}_{\mathrm{wC}2}={\mathrm{r}}_{\mathrm{S}}+{\mathrm{r}}_{\mathrm{C}2}+{\mathrm{h}}_{\mathrm{a}}-{\mathrm{h}}_{\mathrm{f}}.$$

The pitch pressure angle for such wheels is:

80 $${\mathrm{\alpha }}_{\mathrm{wQ}}=\mathrm{arccos}\left(\frac{{\mathrm{r}}_{\mathrm{S}}+{\mathrm{r}}_{\mathrm{Q}}}{{\mathrm{a}}_{\mathrm{wQ}}}\cdot \mathrm{cos}{\mathrm{\alpha }}_{\mathrm{p}}\right).$$

Because h_f_ > h_a_, there are worse conditions for interaction between the teeth of the satellite and the teeth of the rotor.

Worse conditions of cooperation include, among others, the appearance of an additional impact force F_im_ of the tooth of the satellite against the tooth of the planet. This force is the effect of the circumferential backlash CB (Fig. 11) and is created when the pressure in the working chambers adjacent to the satellite changes abruptly. So this process takes place for ${\alpha }_{R}=\frac{180}{{n}_{E}}$, ${\alpha }_{R}=\frac{180}{{n}_{E}}+{{\alpha }_{R}}^{\left(C\right)}$, ${\alpha }_{R}=\frac{180}{{n}_{R}}$, ${\alpha }_{R}=\frac{180}{{n}_{R}}+{{\alpha }_{R}}^{\left(C\right)}$ e.t.c. (Fig. 9), where ${{\alpha }_{R}}^{\left(C\right)}$ is given by Eq. (62). The value of the impact force F_im_ of the satellite tooth on the rotor tooth can be calculated according to the formula:

81 $${F}_{im}=4\cdot \frac{1}{t}\cdot \sqrt{CB\cdot {r}_{S}\cdot H\cdot {m}_{S}\cdot {\Delta p}_{S}},$$

where t—the duration of the impact, H—the height of the satellite, m_S_—the mass of the satellite.

## Parameters of the tested satellite mechanism

The satellite mechanism type 4 × 6 (Fig. 3) with a tooth module 0.6 mm was used in a hydraulic motor (Fig. 2). As already mentioned in “Introduction” section, this mechanism is a scaled copy of the mechanism with module 1.5 mm. The mechanism with modulus 1.5 mm was designed to be manufactured by classical machining methods (chiselling and milling) and using special instruments^47,50^. The pitch line of the rotor consists of circles with radii r_P_ and r_Q_ connected at point T (point T is the point of contact of these circles). The pitch line of the curvature is approximated by fragments of circles with radii r_C1_ and r_C2_^47^ (Fig. 10). The satellite mechanism with a module of 0.6 mm was manaufactured using the wire electrical discharge machining (WEDM) process. Technical data of the satellite mechanism are presented in Table 2. While Table 3 shows the values of the distance between the axes, the values of the rolling pressure angle and the values of the contact ratio, assuming that in the working mechanism there is a change in the distance between the axis of the satellite and the axes of the humps of the rotor and the humps of the curvature.

**Table 2:** Parameters of the tested satellite mechanism.

n_R_	n_E_	b_R_	b_E_	m	H
4	6	45°	30°	0.6	20 mm

z_S_	z_R_	z_E_	r_S_	p_bs_	m_S_
10	44	66	3.0 mm	1.77 mm	4.381 g

r_P_	x_P_	y_P_	r_Q_	x_Q_	y_Q_
202.2 mm	0.0 mm	213.78 mm	6 mm	5.657 mm	5.657 mm

x_T_	y_T_	L_M_	L_m_	h_a_	h_f_
6.895 mm	11.130 mm	5.508 mm	4.859 mm	0.45 mm	0.51 mm

r_C1_	x_C1_	y_C1_	r_C2_	x_C2_	y_C2_
9.333 mm	5.380 mm	9.319 mm	5.898 mm	20.333 mm	11.739 mm

α_p_	a_wQ_	a_wP_	a_wC1_	a_wC2_	ε_rP_
20°	8.995 mm	199.21 mm	6.333 mm	8.899 mm	2.438

ε_rQ_	ε_rC1_	ε_rC2_	z_rQ_*	z_rP_**	z_rC1_***
1.155	2.347	1.152	20	674	31.11

z_rC2_****					
19.66					

*zrQ—the theoretical number of teeth on a wheel of radius r_Q_.

**zrP—the theoretical number of teeth on a wheel of radius r_P_.

***zrC1—the theoretical number of teeth on a wheel of radius r_C1_.

****zrC2—the theoretical number of teeth on a wheel of radius r_C2_.

**Table 3:** Distances between the axes, angles of pressure and contact ratio, assuming the elimination of the tip clearances of the teeth of the satellite and curvature in the working mechanism.

a_wQ_	a_wP_	a_wC1_	a_wC2_	α_wP_	α_wQ_
9.055 mm	199.265 mm	6.273 mm	8.838 mm	20.05	21.02

α_wC1_	α_wC2_	ε_rP_	ε_rQ_	ε_rC1_	ε_rC2_
18.43	18.90	2.338	1.058	2.450	1.254

The mechanism was made of NIMAX steel^72^. The parameters of this steel are given in Table 4.

**Table 4:** Basic parametres of the NIMAX steel^72^.

Density	Young module E	Poisson number v	Surface hardness after gas nitriding
7900 kg/m^3^	2.05 × 10^11^ Pa	0.3	950 MHV

Hardness	Yield strength Rp_0.2_	Tensile strength R_m_	Depth after gas nitriding
~ 370 HB	785 MPa	1265 MPa	0.25 mm

For nitrided NIMAX steel, it can be assumed that σ_Hlim_ ≈ 1250 MPa^57^.

**Figure 12:**
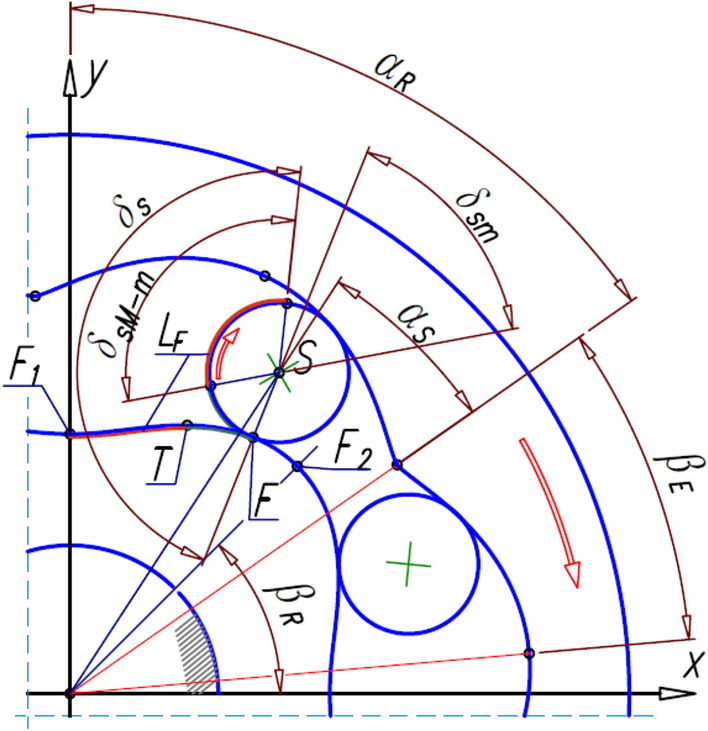
Angles in the tested mechanism and point T.

The coordinates (x,y) of the pitch line of the rotor can be calculated using formula (Fig. 12):

1. from point F_1_ to point T:
  82 $$\mathrm{x}=\frac{\mathrm{cot}{\mathrm{\alpha }}_{\mathrm{R}}\cdot {\mathrm{y}}_{\mathrm{p}}-\sqrt{\mathrm{cot}{\mathrm{\alpha }}_{\mathrm{R}}\cdot {\mathrm{y}}_{\mathrm{p}}\cdot \left(\mathrm{cot}{\mathrm{\alpha }}_{\mathrm{R}}\cdot {\mathrm{y}}_{\mathrm{p}}+2\cdot \left({{\mathrm{y}}_{\mathrm{P}}}^{2}-{{\mathrm{r}}_{\mathrm{P}}}^{2}\right)\right)}}{{\mathrm{cot}}^{2}{\mathrm{\alpha }}_{\mathrm{R}}+1},$$
  83 $$\mathrm{y}=\mathrm{cot}{\mathrm{\alpha }}_{\mathrm{R}}\cdot {\mathrm{x}}_{\mathrm{R}}.$$
2. from point T to point F2:
  84 $$x=\frac{{x}_{Q}+{\cot}{\upalpha }_{R}\cdot {y}_{Q}+\sqrt{{\left(\mathit{cot}{\alpha }_{R}\cdot {y}_{Q}+{x}_{Q}\right)}^{2}-\left({\mathit{cot}}^{2}{\alpha }_{R}+1\right)\cdot \left({x}_{Q}+{y}_{Q}-{r}_{Q}\right)}}{{\mathit{cot}}^{2}{\alpha }_{R}+1}.$$

The y-coordinate is calculated according to formula (83).

The relationship between the coordinates of points S and F is as follows. If the point F of contact of the satellite pitch line with the rotor pitch line is between points F_1_ and T, then (Fig. 12):

85 $${\mathrm{x}}_{\mathrm{F}}=\frac{1}{{\mathrm{r}}_{\mathrm{P}}-{\mathrm{r}}_{\mathrm{s}}}\cdot \left({\mathrm{r}}_{\mathrm{P}}\cdot {\mathrm{x}}_{\mathrm{s}}-{\mathrm{r}}_{\mathrm{s}}\cdot {\mathrm{x}}_{\mathrm{p}}\right),$$

86 $${\mathrm{y}}_{\mathrm{F}}=\frac{1}{{\mathrm{r}}_{\mathrm{P}}-{\mathrm{r}}_{\mathrm{s}}}\cdot \left({\mathrm{r}}_{\mathrm{P}}\cdot {\mathrm{y}}_{\mathrm{s}}-{\mathrm{r}}_{\mathrm{s}}\cdot {\mathrm{y}}_{\mathrm{p}}\right),$$

87 $${\mathrm{x}}_{\mathrm{s}}=\frac{{\mathrm{x}}_{\mathrm{p}}+{\mathrm{a}}_{\mathrm{s}}\cdot {\mathrm{y}}_{\mathrm{p}}}{{{\mathrm{a}}_{\mathrm{s}}}^{2}+1}-\sqrt{{\left(\frac{{\mathrm{x}}_{\mathrm{p}}+{\mathrm{a}}_{\mathrm{s}}\cdot {\mathrm{y}}_{\mathrm{p}}}{{{\mathrm{a}}_{\mathrm{s}}}^{2}+1}\right)}^{2}-\frac{{{\mathrm{x}}_{\mathrm{p}}}^{2}+{{\mathrm{y}}_{\mathrm{p}}}^{2}-{\left({\mathrm{r}}_{\mathrm{p}}-{\mathrm{r}}_{\mathrm{s}}\right)}^{2}}{{{\mathrm{a}}_{\mathrm{s}}}^{2}+1}},$$

88 $${\mathrm{y}}_{\mathrm{s}}={\mathrm{a}}_{\mathrm{s}}\cdot {\mathrm{x}}_{\mathrm{s}},$$

where

89 $${\mathrm{a}}_{\mathrm{s}}=\mathrm{cot}\left({\mathrm{\alpha }}_{\mathrm{R}}-{\mathrm{\alpha }}_{\mathrm{s}}\right).$$

If point F is between points T and F_2_, then the relationship between the coordinates of points S and F is as follows (Fig. 12):

90 $${\mathrm{x}}_{\mathrm{F}}=\frac{1}{{\mathrm{r}}_{\mathrm{Q}}+{\mathrm{r}}_{\mathrm{s}}}\cdot \left({\mathrm{r}}_{\mathrm{Q}}\cdot {\mathrm{x}}_{\mathrm{s}}+{\mathrm{r}}_{\mathrm{s}}\cdot {\mathrm{x}}_{\mathrm{Q}}\right),$$

91 $${\mathrm{y}}_{\mathrm{F}}=\frac{1}{{\mathrm{r}}_{\mathrm{Q}}+{\mathrm{r}}_{\mathrm{s}}}\cdot \left({\mathrm{r}}_{\mathrm{Q}}\cdot {\mathrm{y}}_{\mathrm{s}}+{\mathrm{r}}_{\mathrm{s}}\cdot {\mathrm{y}}_{\mathrm{Q}}\right),$$

92 $${\mathrm{x}}_{\mathrm{S}}=\frac{{\mathrm{x}}_{\mathrm{Q}}+{\mathrm{a}}_{\mathrm{s}}\cdot {\mathrm{y}}_{\mathrm{Q}}}{{{\mathrm{a}}_{\mathrm{s}}}^{2}+1}+\sqrt{{\left(\frac{{\mathrm{x}}_{\mathrm{Q}}+{\mathrm{a}}_{\mathrm{s}}\cdot {\mathrm{y}}_{\mathrm{Q}}}{{{\mathrm{a}}_{\mathrm{s}}}^{2}+1}\right)}^{2}-\frac{{{\mathrm{x}}_{\mathrm{Q}}}^{2}+{{\mathrm{y}}_{\mathrm{Q}}}^{2}-{\left({\mathrm{r}}_{\mathrm{Q}}+{\mathrm{r}}_{\mathrm{s}}\right)}^{2}}{{{\mathrm{a}}_{\mathrm{s}}}^{2}+1}.}$$

The values of y_S_ and a_S_ should be calculated according to formulae (88) and (89) respectively.

If the point F coincides with the point T (i.e. x_F_ = x_T_ i y_F_ = y_T_) then the coordinates of the satellite centre are as follows:

93 $${\mathrm{x}}_{\mathrm{S}}=\left(1-\frac{{r}_{s}}{{r}_{P}}\right)\cdot {x}_{T}+\frac{{r}_{s}}{{r}_{P}}\cdot {x}_{p},$$

94 $${\mathrm{y}}_{\mathrm{S}}=\left(1-\frac{{r}_{s}}{{r}_{P}}\right)\cdot {y}_{T}+\frac{{r}_{s}}{{r}_{P}}\cdot {y}_{p},$$

and the curvature must make a rotation through an angle:

95 $${\mathrm{\alpha }}_{\mathrm{T}}=\left(1+\frac{{\mathrm{n}}_{\mathrm{R}}}{{\mathrm{n}}_{\mathrm{E}}}\right)\cdot \mathrm{arctan}\left(\frac{{\mathrm{x}}_{\mathrm{S}}}{{\mathrm{y}}_{\mathrm{S}}}\right).$$

For the considered mechanism, this is the angle α_T_ = 33.80°.

### The rotation angles of the satellite and the number of teeth contacts in the mechanism

For one full revolution of the rotor, i.e. for α_R_ = 360°:

- the angle of rotation of the satellite about its axis is δ_S(αR = 360°)_ = 1584°;
- the satellite moves relative to the curvature by an angle α_S_ = 144°;
- the angle α_S_ = 144° corresponds to the length L_E_ = 49.73 mm of the curvature pitch line;
- the number of contacts of each tooth of the satellite with:
  the teeth of the rotor (and hence the number of revolutions of the satellite about its own axis) is i_SR_ = 4.4;
  the teeth of the curvature (and hence the number of revolutions of the satellite about its own axis) is i_SE_ = 2.6;
- the total number of contacts of the teeth of the satellite with the teeth of the rotor and the curvature is i_S_ = 7;
- the number of contacts of each rotor tooth with the satellite tooth is i_RTS_ = 6;
- the number of contacts of each curvature tooth with the satellite tooth is i_ETS_ = 4.

However, for the satellite to make one complete revolution in relation to the curvature, i.e. α_S_ = 360°, the shaft (rotor) must make i_RE_ = 2.5 revolutions.

In the motor working at n = 1500 rpm, in 1 min:

- the number of contacts of each tooth of the satellite with the teeth of the rotor:
  96 $${\mathrm{i}}_{\mathrm{SR}(\mathrm{n})}=\mathrm{n}\cdot {\mathrm{i}}_{\mathrm{SR}}=6600,$$
- the number of contacts of each tooth of the satellite with the teeth of the curvature:
  97 $${\mathrm{i}}_{\mathrm{SE}(\mathrm{n})}=\mathrm{n}\cdot {\mathrm{i}}_{\mathrm{SE}}=3900,$$
- the number of contacts of the teeth of the satellite with the teeth of the rotor and the curvature:
  98 $${\mathrm{i}}_{\mathrm{S}(\mathrm{n})}=\mathrm{n}\cdot {\mathrm{i}}_{\mathrm{S}}=\mathrm{10,500},$$
- the number of contacts of each tooth of the rotor with the tooth of the satellites:
  99 $${\mathrm{i}}_{\mathrm{RTS}(\mathrm{n})}=\mathrm{n}\cdot {\mathrm{i}}_{\mathrm{RTS}}=9600,$$
- the number of contacts of each curvature tooth with the tooth of the satellites:
  100 $${\mathrm{i}}_{\mathrm{ETS}(\mathrm{n})}=\mathrm{n}\cdot {\mathrm{i}}_{\mathrm{ETS}}=6000.$$

### Accelerations of the satellite

**Figure 13:**
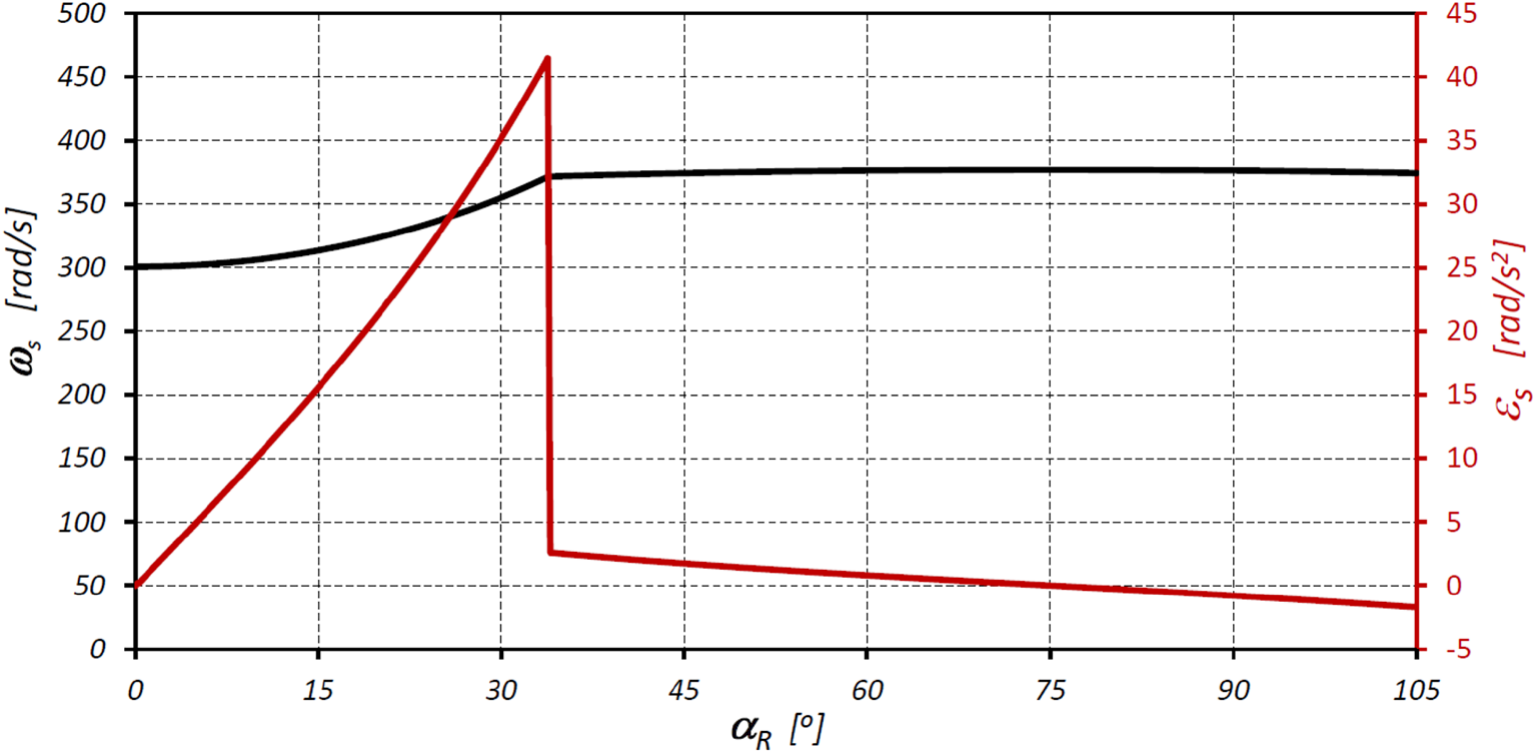
Characteristics of the angular velocity ω_S_ and angular acceleration ε_S_ of the satellite as a function of the angle of rotation of the shaft (rotor) α_R_.

**Figure 14:**
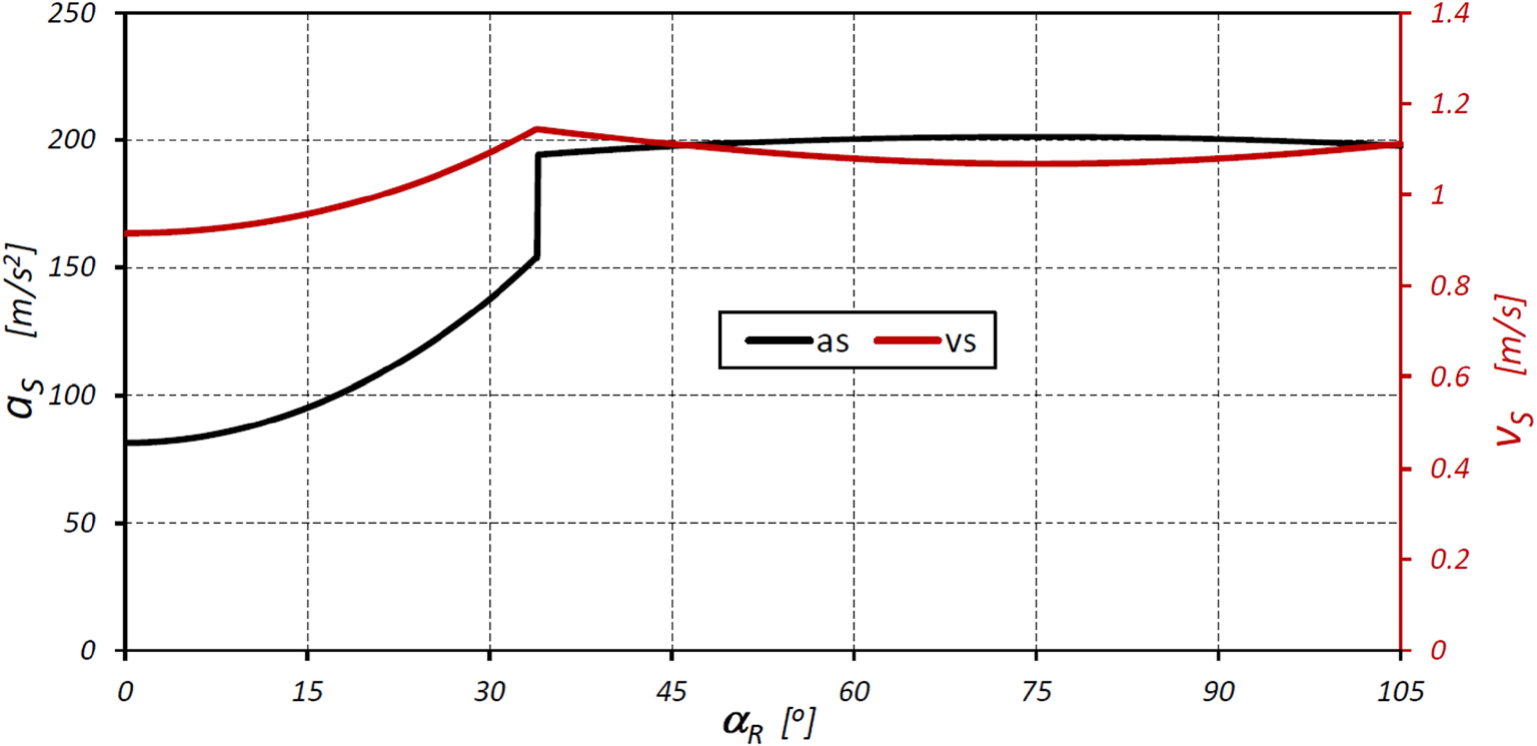
Characteristics of the linear velocity v_S_ and linear acceleration of the satellite as a function of the angle of rotation of the shaft (rotor) α_R_.

The characteristics of the angular velocity ω_S_ and the angular acceleration ε_S_ of the satellite in the considered satellite mechanism are presented in Fig. 13, while in Fig. 14 the characteristics of the linear accelerations of the satellite are presented.

### Tooth loads—forces at points E* and F*

**Figure 15:**
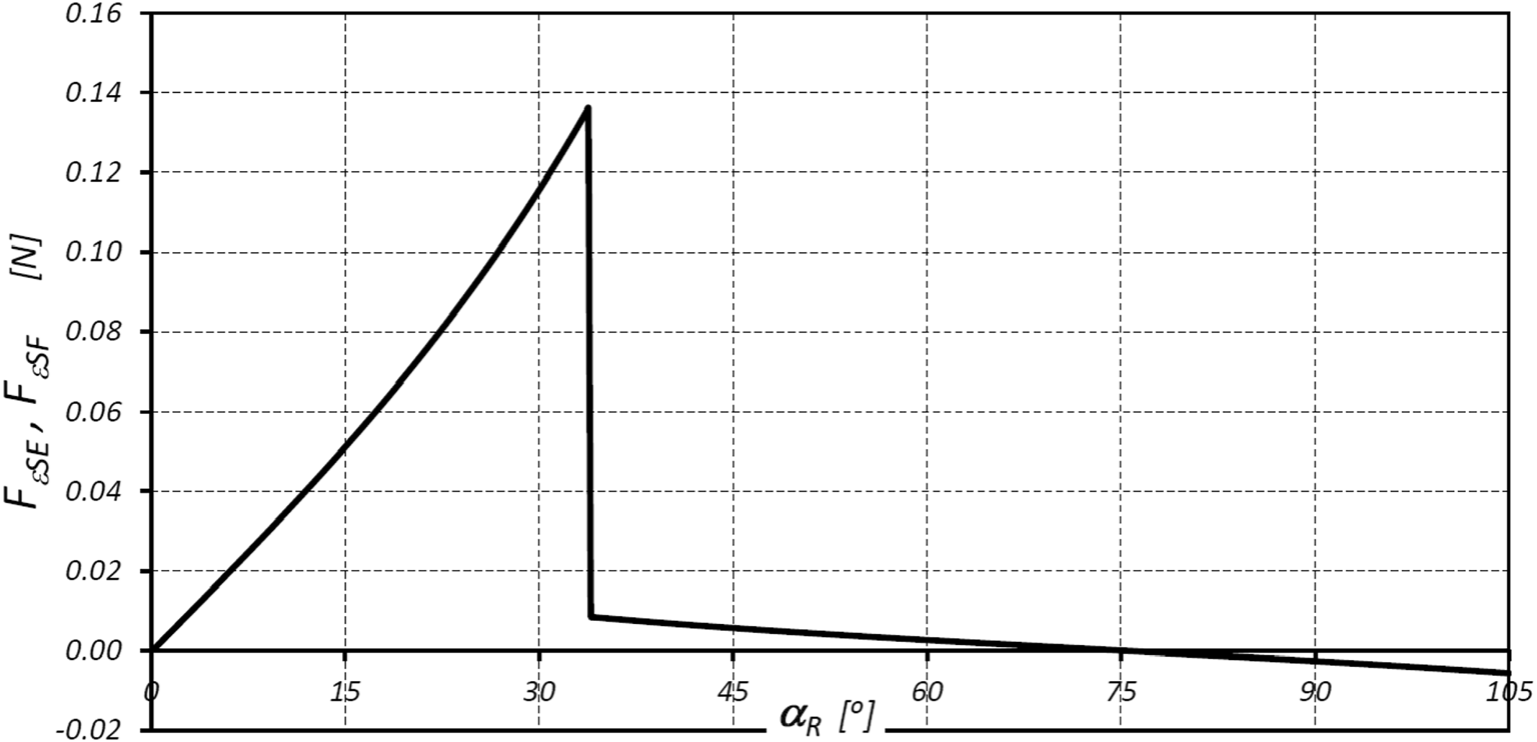
Characteristics of the forces F_εSE_ and F_εSF_.

**Figure 16:**
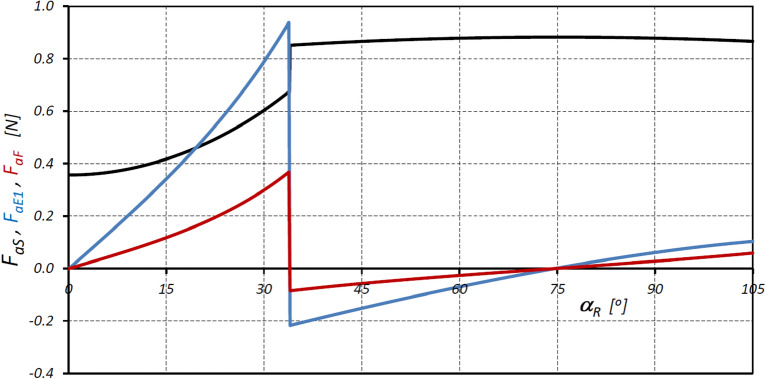
Characteristics of the forces F_aS_, F_aE1_ and F_aF_.

The values of the forces acting at points E* and F* (loading of the teeth) and resulting from the accelerations of the satellite, are shown in Figs. 15 and 16.

**Figure 17:**
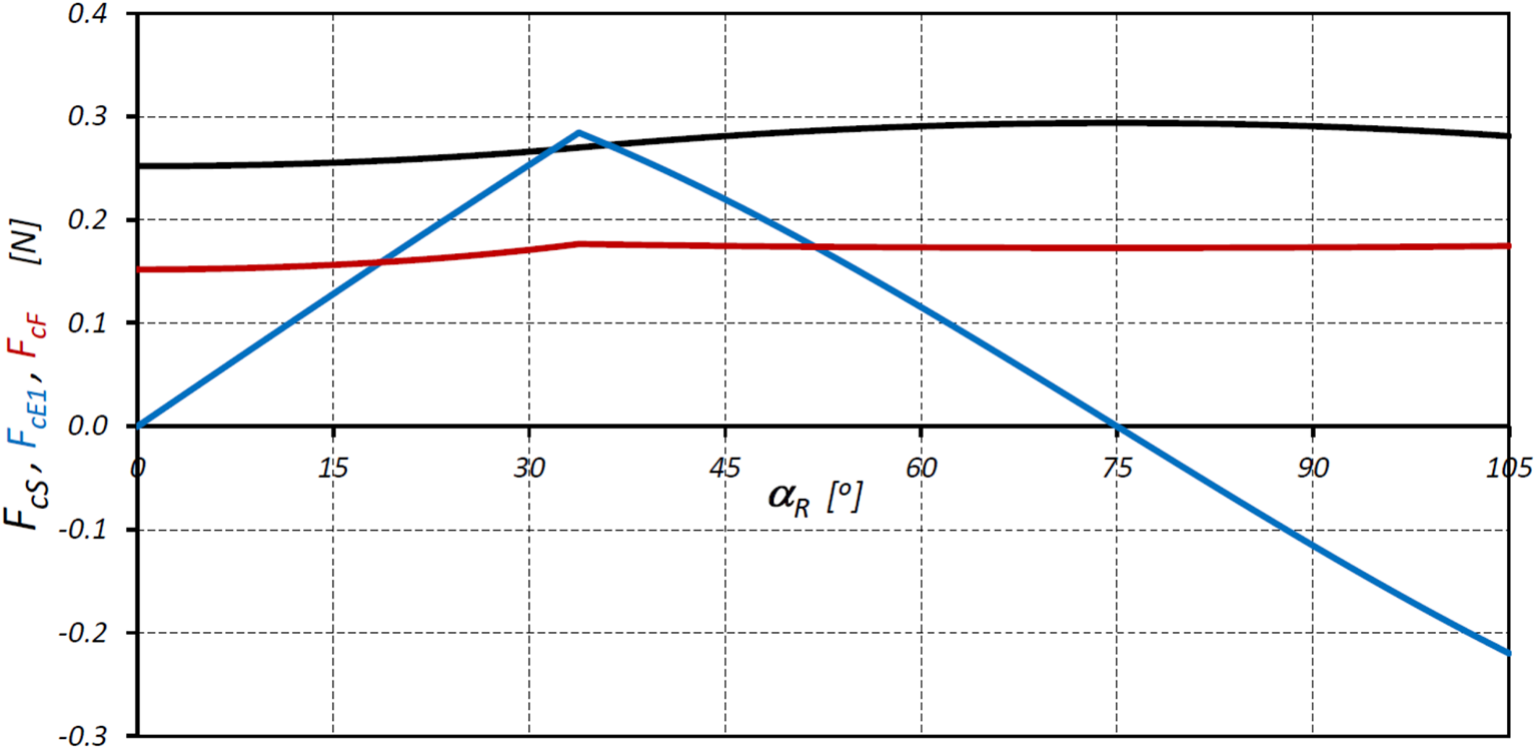
Characteristics of the centrifugal force F_cS_ and the forces F_cE1_ and F_cF_ at points E* and F*.

The characteristics of the centrifugal force F_cS_ acting on the satellite and the values of the forces F_cE1_ and F_cF_ corresponding to this force at points E* and F* of the satellite mechanism under consideration are shown in Fig. 17. And Fig. 17 shows the characteristics of the force F_∆p_ at points E* and F*, calculated according to formula (64).

**Figure 18:**
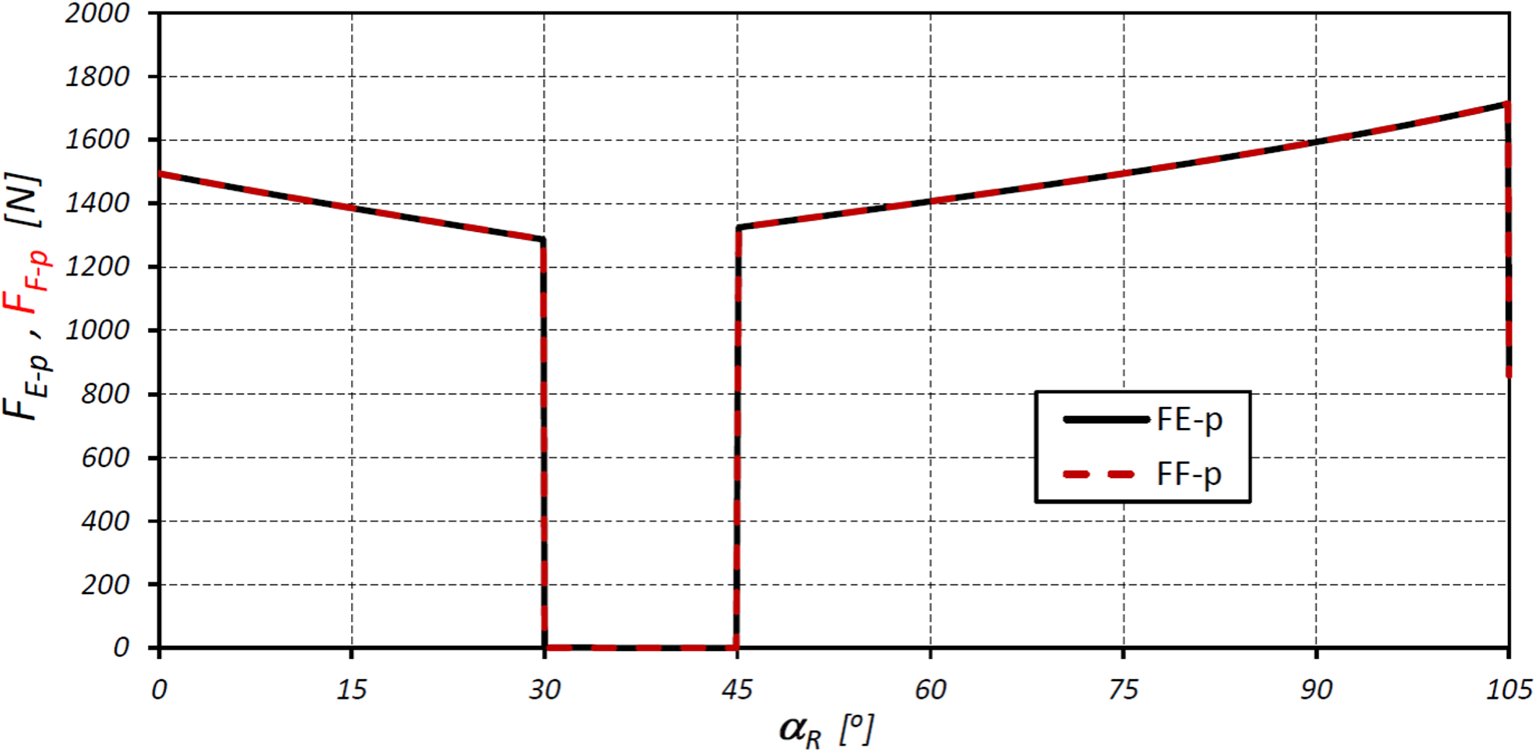
Characteristics of the circumferential forces F_E-p_ and F_F-p_ at the points E* and F*.

The characteristics of the total forces F_E-p_ and F_F-p_ loading the teeth of the interacting elements of the satellite mechanism at points E* and F* are shown in Fig. 18.

### Normal stress on the contact surface of the teeth and permissible stress

The satellite mechanism is not a typical toothed gear mechanism. The satellite is not mounted on a bearing shaft and the rotor and curvature are elements with non-circular gear rims. Therefore, at the present stage of research, it is not possible to give the exact values of the coefficients K and Z from the formulae (73) and (75). It was therefore assumed that the pressures in the contact of mating teeth are calculated for:

- Two values of the coefficient K, i.e. for K = 1 (mechanism elements do not rotate and the teeth of the mechanism elements are loaded only with the force resulting from the pressure difference in the working chambers) and for the approximate value of K = 3.
- Two extreme values of the coefficient Z, i.e. for Z = 0.96 and Z = 1.8.

The calculations of the contact stresses at points E and F were carried out for two cases, namely for:

- Perfect cooperation of the satellite with the rotor and the curvature (i.e. without changing the axis distance).
- For the case of elimination of the tip clearance of the cooperating teeth of the satellite with the curvature.

A summary of the results for the above cases for different rotation angles α_R_ of the rotor can be found in Tables 5 and 6 respectively. Whereas the values of permissible stresses are presented in Table 7.

**Table 5:** Values of normal stress σ_N_ (in MPa) at the points E* and F* (Fig. 9).

Contact point	α_R_ [deg]	∆p_S_ = 25 MPa		∆p_S_ = 5 MPa	
		K = 1	K = 3	K = 1	K = 3
E* (Fig. 9)	0	1211.5	2098.4	537.3	930.7
	30	1124.4	1947.6	498.5	863.5
	45	1871.6	3241.7	830.4	1438.3
	105	2129.4	3688.2	944.8	1636.6
F* (Fig. 9)	0	1994.5	3454.6	884.8	1532.5
	30	1319.3	2285.2	584.2	1011.9
	45	1338.8	2318.8	594.0	1028.8
	105	1523.0	2637.9	675.5	1170.0

**Table 6:** Values of normal stress σ_N_ (in MPa) at the points E* and F* (Fig. 9)—assuming the clearance of the mating teeth of the satellite and curvature (in the working mechanism) is deleted.

Contact point	α_R_ [deg]	∆p_S_ = 25 MPa		∆p_S_ = 5 MPa	
		K = 1	K = 3	K = 1	K = 3
E* (Fig. 9)	0	1247.9	2126.4	553.5	958.6
	30	1158.2	2006.0	513.5	889.4
	45	1858.5	3219.0	824.6	1428.2
	105	2114.4	3662.3	938.2	1625.1
F* (Fig. 9)	0	2012.4	3585.6	892.7	1546.2
	30	1328.7	2301.3	588.3	1019.0
	45	1348.3	2335.2	598.2	1036.0
	105	1533.8	2656.6	680.3	1178.3

**Table 7:** Permissible stress σ_Hlim_ (in MPa).

		Z	
		0.96	1.8
SH	1	1200	2250
	1.3	923.1	1730.8

If the phenomenon of the impact of the satellite tooth on the rotor tooth is taken into account, the value of the pressure force of the satellite tooth on the rotor tooth will increase by F_im_ and the value of stresses σ_N_ at the point of contact of these teeth will increase. The values of the Fim force are presented in Table 8, and the stress values in Table 9.

**Table 8:** Impact force F_im_ values (in N) of the satellite tooth against the rotor tooth.

CB [mm]	t [s]	∆p_S_ = 25 MPa	∆p_S_ = 5 MPa
0.05	0.001	72.5	32.4
	0.010	7.25	3.24
0.10	0.001	102.5	45.9
	0.010	10.25	4.59

**Table 9:** Values of normal stress σ_N_ (in MPa) at the point F* (in MPa) assuming the impact of the satellite tooth against the rotor tooth (for t = 0.001 s).

CB [mm]	aR [deg]	∆p_S_ = 25 MPa		∆p_S_ = 5 MPa	
		K = 1	K = 3	K = 1	K = 3
0.05	30	1190.4	2061.8	545.4	944.7
	45	1908.7	3305.9	874.4	1514.4
	105	2158.9	3738.9	982.3	1701.3
0.10	30	1203.4	2084.4	558.1	966.6
	45	1929.1	3341.2	894.2	1548.7
	105	2176.7	3770.2	999.9	1731.9

## Conclusions

**Figure 19:**
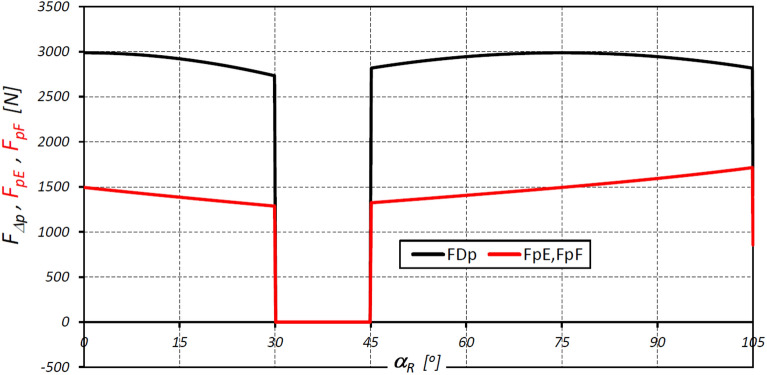
Characteristics of the force F_∆p_ and the forces F_pE_ and F_pF_ at the points E* and F*.

The results of the analyses have shown that in the satellite mechanism:

- The number of contacts of each rotor tooth with a satellite tooth is 1.5 times larger (i_RTS_ = 6 and i_ETS_ = 4) than the number of contacts of each curvature tooth with the satellites tooth.
- The accelerations of the satellite and the corresponding forces loading the teeth are a non-linear function of the rotation angle of the rotor (Figs. 13, 16).
- The highest values of the forces acting on the teeth of the satellite mechanism result from the pressure difference ∆p_S_ acting on the satellite. The highest value of these forces occurs for α_R_ = 105°, i.e. when the working chamber C reaches the minimum volume V_C-min_ (Fig. 9e). The value of the forces F_pE_ and F_pF_ for α_R_ = 105° and for the pressure difference ∆p_S_ in two adjacent working chambers of 25 MPa is 1714 N (Fig. 19).
- The values of F_pE_ and F_pF_ within the angle of rotation of the shaft ${\mathrm{\alpha }}_{\mathrm{R}}\in \left(\frac{180}{{\mathrm{n}}_{\mathrm{E}}}={30}^{^\circ };\frac{180}{{\mathrm{n}}_{\mathrm{R}}}={45}^{^\circ }\right)$ are 0N (Fig. 19). Within this angular range, the working chambers adjacent to the satellite are in the same phase (emptying—Fig. 9c).
- The forces F_eSE_, F_eSF_, F_aE1_ and F_aF_ loading the teeth resulting from satellite accelerations and forces F_cE1_ and F_cF_ due to the centrifugal force F_c_ have very low values of less than 1 N (Figs. 15, 16, 17). The highest values of these forces occur for the rotor rotation angle α_T_ = 33.80° (point T is the contact point of the satellite with the rotor—Fig. 12). After passing the point T, the values of the forces F_eSE_, F_eSF_, F_aE1_ and F_aF_ decrease rapidly due to changes in the geometric parameters of the rotor. The rapid changes in these forces do not have a significant effect on the value of the stress at the contact point of the interacting teeth due to their very small values.
- The forces F_eSE_, F_eSF_, F_aE1_, F_aF_ and forces F_cE1_ and F_cF_ can be omitted from the consideration of stresses in the teeth contact due to their low values compared to the forces F_pE_ and F_pF_.
- There is a shift of the satellite towards the curvature within the limits of the tip clearances and circumferential backlash of the mating teeth;
- At the moment when there is a step change in pressure in the working chambers (for the presented mechanism α_R_ = 30°, 45°, 105°, 120° etc. (Fig. 9)) the tooth of the satellite hits the tooth of the rotor with the force F_im_, the value of which depends especially on the value of clearance CB and may exceed the force F_pF_ resulting directly from the pressure difference ∆p_S_ (Table 8).
- In ideal satellite mechanism (without clearances) the stresses in the contact of the interacting teeth do not exceed the value of allowable stresses at ∆p_S_ = 25 MPa only in the most optimistic calculation variant (for K = 1, SH = 1 and Z = 1.8).
- In a real satellite mechanism, in which the impact force F_im_ of the satellite tooth against the planet tooth occurs, the stresses in the contact of the cooperating satellite teeth with the rotor teeth are about 4% higher (for ∆p_S_ = 25 MPa) than the stresses in the contact of the cooperating satellite teeth with the curvature teeth (see the results in Tables 6, 9).

In addition, based on the results of the analyzes carried out, it can be concluded that the following factors are the reason for the rapid wear of the rotor teeth (Fig. 3):

- The tip clearances and circumferential backlash of the cooperating teeth, as a result of which an undesirable impact force F_im_ of the satellite tooth against the rotor tooth is created.
- During the operation of the satellite mechanism, the clearance probably increases (as a result of wear), which increases the impact force F_im_.
- To large working pressure of the satellite motor (the working pressure of 25 MPa is too large for satellite mechanism).
- High rotational speed of the rotor. As shown in “The rotation angles of the satellite and the number of teeth contacts in the mechanism” section, at the rotation speed of the motor shaft n = 1500 rpm, the number of contacts of each rotor tooth with the tooth of the satellites during one minute is as much as 9600. In 1 h there are 576,000 contacts and in 10 h at least 5.76 million contacts. Considering the dynamic changes in the load on the teeth, it can be assumed that the satellite mechanism will be destroyed in a relatively short operating time.

Comparing the calculation results, given in Tables 5 and 7, it can be presumed that the wear of the rotor teeth will be significantly reduced when:

- The satellite mechanism will be precisely made, i.e. with minimal tip clearances and with minimal circumferential backlash (CB < 0.08 × module).
- The motor will be operated with such a torque load that the pressure difference ∆p_i_ in the working chambers does not exceed 5 MPa.

In summary, satellite positive displacement machines (pumps, motors) should operate at low speed and at most an average working pressure. For example, if the permissible speed of the hydraulic motor shaft is 100 rpm, the number of contacts of each rotor tooth with the satellite tooth will be only 38,400 within 1 h.

## Data Availability

The datasets used and/or analysed during the current study are available from the corresponding author on reasonable request.
